# A newly introduced salt bridge cluster improves structural and biophysical properties of *de novo* TIM barrels

**DOI:** 10.1002/pro.4249

**Published:** 2021-12-16

**Authors:** Sina Kordes, Sergio Romero‐Romero, Leonie Lutz, Birte Höcker

**Affiliations:** ^1^ Department of Biochemistry University of Bayreuth Bayreuth Germany

**Keywords:** (β/α)_8_ barrel, *de novo* protein design, DeNovoTIMs, protein folding, protein stability, salt bridge cluster, TIM barrel

## Abstract

Protein stability can be fine‐tuned by modifying different structural features such as hydrogen‐bond networks, salt bridges, hydrophobic cores, or disulfide bridges. Among these, stabilization by salt bridges is a major challenge in protein design and engineering since their stabilizing effects show a high dependence on the structural environment in the protein, and therefore are difficult to predict and model. In this work, we explore the effects on structure and stability of an introduced salt bridge cluster in the context of three different *de novo* TIM barrels. The salt bridge variants exhibit similar thermostability in comparison with their parental designs but important differences in the conformational stability at 25°C can be observed such as a highly stabilizing effect for two of the proteins but a destabilizing effect to the third. Analysis of the formed geometries of the salt bridge cluster in the crystal structures show either highly ordered salt bridge clusters or only single salt bridges. Rosetta modeling of the salt bridge clusters results in a good prediction of the tendency on stability changes but not the geometries observed in the three‐dimensional structures. The results show that despite the similarities in protein fold, the salt bridge clusters differently influence the structural and stability properties of the *de novo* TIM barrel variants depending on the structural background where they are introduced.

## Abstract 2

PDB Code(s): 7OSU, 7OT7, 7OSV, 7OT8 and 7P12;

**Abbreviations**
CD: circular dichroism
DSC: differential scanning calorimetry
D_[1/2]_
: midpoint urea unfolding concentration
IF: intrinsic fluorescence
MALS: multi angle light scattering
REU: Rosetta energy unit
SEC: size exclusion chromatography
*T*_m_
: midpoint of thermal unfolding
Δ*C*_*P*_
: change in heat capacity
Δ*G*_25°C_
: change in Gibbs free energy at 25°C
Δ*H*
: change in enthalpy

## 1 INTRODUCTION

Protein stability is a fundamental biological attribute that modulates the delicate balance among protein evolvability, expression, solubility, structure, and function.^1^, ^2^, ^3^ It results from the accumulated balance of forces and interactions between protein and solvent that determines whether the folded conformation is stable over other nonfunctional competing states. The central role of proteins in the chemistry of life, as well as their increasing application in basic and applied research, makes an understanding of protein stability highly relevant.

The information obtained about the forces that fine‐tune protein stability come from numerous studies on natural proteins and have led to the possibility to design proteins from scratch. Those computationally designed proteins differ significantly in sequence and structure from naturally occurring proteins, providing new information to gain a deeper understanding of the relationship between sequence, structure, and stability.^4^

Several strategies to increase protein stability have been explored such as rearrangement of hydrogen‐bond networks, introduction of salt bridges, improving of hydrophobic cores, or incorporation of covalent bonds.^5^, ^6^, ^7^, ^8^, ^9^ Among them, the prediction and engineering of salt bridges is challenging, due to their high dependence on the structural environment of the protein and the requirement of accurate geometries.^1^, ^10^ Consequently, it is very difficult to estimate the energetic contribution to stability caused by a salt bridge due to a delicate balance of destabilizing desolvation energy and stabilizing interactions.^11^

A salt bridge can be defined as an ion‐pair interaction between two residues of opposite charge with a distance below 4 Å that combines two noncovalent interactions, hydrogen bonding and ionic bonding.^12^ This type of interaction plays an important role in defining protein structure, function and stability^11^, ^13^, ^14^, ^15^, ^16^ and has been a valuable strategy in protein engineering to stabilize different proteins and calculate their energetic contributions.^17^, ^18^, ^19^, ^20^, ^21^ In addition, the interaction of one basic residue with multiple acidic residues form clustered or networked salt bridges, which are of special interest due to their complexity and important contribution to protein stability.^10^, ^22^, ^23^

Among all protein architectures, the TIM barrel is one of the most common folds in nature, as one‐tenth of the known proteins adopt this topology and it is found in five out of seven enzyme classes.^24^, ^25^ This ubiquitous and versatile topology has been an important model system to study not only the stability, structure, and function relationships but also for *de novo* protein design. Previously, we demonstrated that increasing the hydrophobic clusters of the first *de novo* TIM barrel sTIM11^26^ resulted in a highly‐stable collection of TIM barrels, which we called DeNovoTIMs.^27^ In the work presented here, we explore the effects on structure and stability when introducing a salt bridge cluster into members of the DeNovoTIM collection.

## 2 RESULTS AND DISCUSSION

### 2.1 *Introducing a salt bridge cluster into different* de novo *TIM barrels*

**FIGURE 1:**
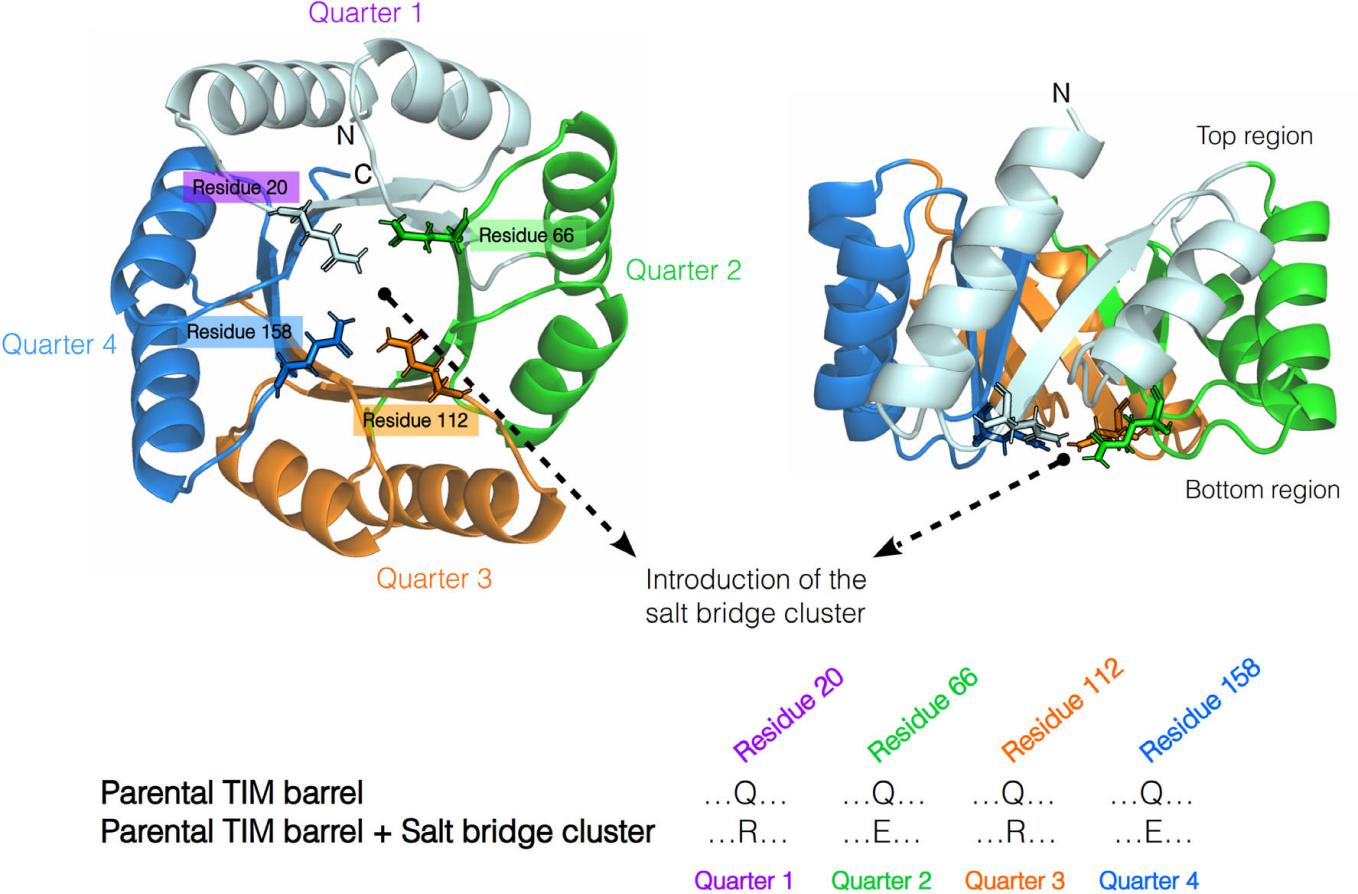
Strategy to introduce a salt bridge cluster in *de novo* TIM barrels. Crystal structure of sTIM11noCys is shown with residues 20, 66, 112, and 158 highlighted as sticks, which were used for the introduction of the salt bridge cluster. It was added in the *de novo* TIM barrels replacing Q20 and Q112, belonging to the first and third quarters, by arginine residues, and Q66 and Q158, from the second and fourth quarters, by glutamic acid residues

The effects of introducing a salt bridge cluster were inspired by the presence of a similar cluster in the natural HisF TIM barrel, a subunit of the imidazole glycerophosphate synthase (IGPS),^28^, ^29^ and its observed influence in stabilizing this fold.^30^, ^31^ Since the salt bridge cluster was intended to be evaluated without affecting the previous stabilized regions in our DeNovoTIM collection, we focused on the internal core of the barrel to introduce the salt bridge network. Therefore, considering the environmental and geometrical descriptors of the most common salt bridges found in natural proteins,^10^ we found that at the internal core of the TIM barrel, and specifically on the bottom part of it, 4 symmetry‐related glutamine residues were suitable to introduce the intended salt bridge cluster. The four residues were alternatively mutated to Arg and Glu in the four quarters as indicated in Figure 1 and Table S1.

We explored the effects of the salt bridge cluster in the context of three different *de novo* TIM barrels previously reported as DeNovoTIM collection,^27^ all designed using a computational fixed‐backbone and modular approach to improve the hydrophobic packing: *sTIM11noCys*, the cysteine‐free variant of sTIM11 (the first validated *de novo* TIM barrel) without any extra stabilizing mutations as present in DeNovoTIMs; *DeNovoTIM6*, with stabilizing mutations in the bottom region of the peripheral core located between the outer face of the β‐strands and the internal face of the α‐helices; and *DeNovoTIM13*, with stabilizing mutations in the bottom and top regions of the peripheral core (Table S1). The TIM‐barrel architecture among the three proteins is conserved with an RMSD <1.5 Å. The main differences are related to the size and packing of the hydrophobic clusters due to the introduced mutations.^27^ Salt bridge variants derived from parental proteins (named as the original design plus the suffix‐SB) were biophysically and structurally characterized as follows.

### 2.2 Salt bridge cluster variants are soluble monomeric and well‐folded TIM barrels

All salt bridge variants were expressed and purified to homogeneity in high amounts with similar yields to the parental proteins. Just as the parental proteins DeNovoTIM6‐SB and DeNovoTIM13‐SB show about 15% dimer in the preparative size exclusion chromatography (SEC). Multi angle light scattering (MALS) measurements of the monomer peak for both proteins at different concentrations revealed no concentration dependent dimerization in the range up to 5 mg ml^−1^. Additionally, the dimer was analyzed and appeared to be stable. All further experiments were done with the monomeric fraction. The molecular weight of all three proteins was determined using MALS and verified as monomers (Figure S1 and Table S2). In contrast to DeNovoTIM13, which showed a tendency for aggregation after purification, DeNovoTIM13‐SB did not aggregate in the observed time frame, probably due to the thermal‐unfolding reversibility which is not present in DeNovoTIM13.

Circular dichroism (CD) analysis indicated well folded proteins with a mixed α/β secondary structure composition similar to the parental proteins (Figure S2, Table S2). For sTIM11noCys‐SB a higher signal at about 222 nm is observed hinting at an increase in the α‐helical fraction. For DeNovoTIM13‐SB additionally the peak at 208 nm is more pronounced showing gain of overall secondary structure. In contrast, DeNovoTIM6‐SB shows an overall decrease of the signal and a more pronounced signal at 208 nm. Deconvolution of the far‐UV CD spectra displays slight differences in the secondary structure contents (Table S2), which is confirmed by the three‐dimensional structure analysis as discussed below. For sTIM11noCys‐SB and DeNovoTIM13‐SB a decrease of random coil connected with an increase of secondary structure content is calculated. In contrast, the deconvolution of DeNovoTIM6‐SB CD spectrum indicates a similar content of random coil but with differences in the ɑ‐helix and β‐sheet composition (Table S2). These data confirm well folded proteins on the basis of their spectroscopy attributes without large structural changes upon the introduction of the salt bridge cluster. To follow up on this, their folding stability behavior was studied by thermal and chemical unfolding experiments.

### 2.3 Thermostability is maintained in the salt bridge variants

**FIGURE 2:**
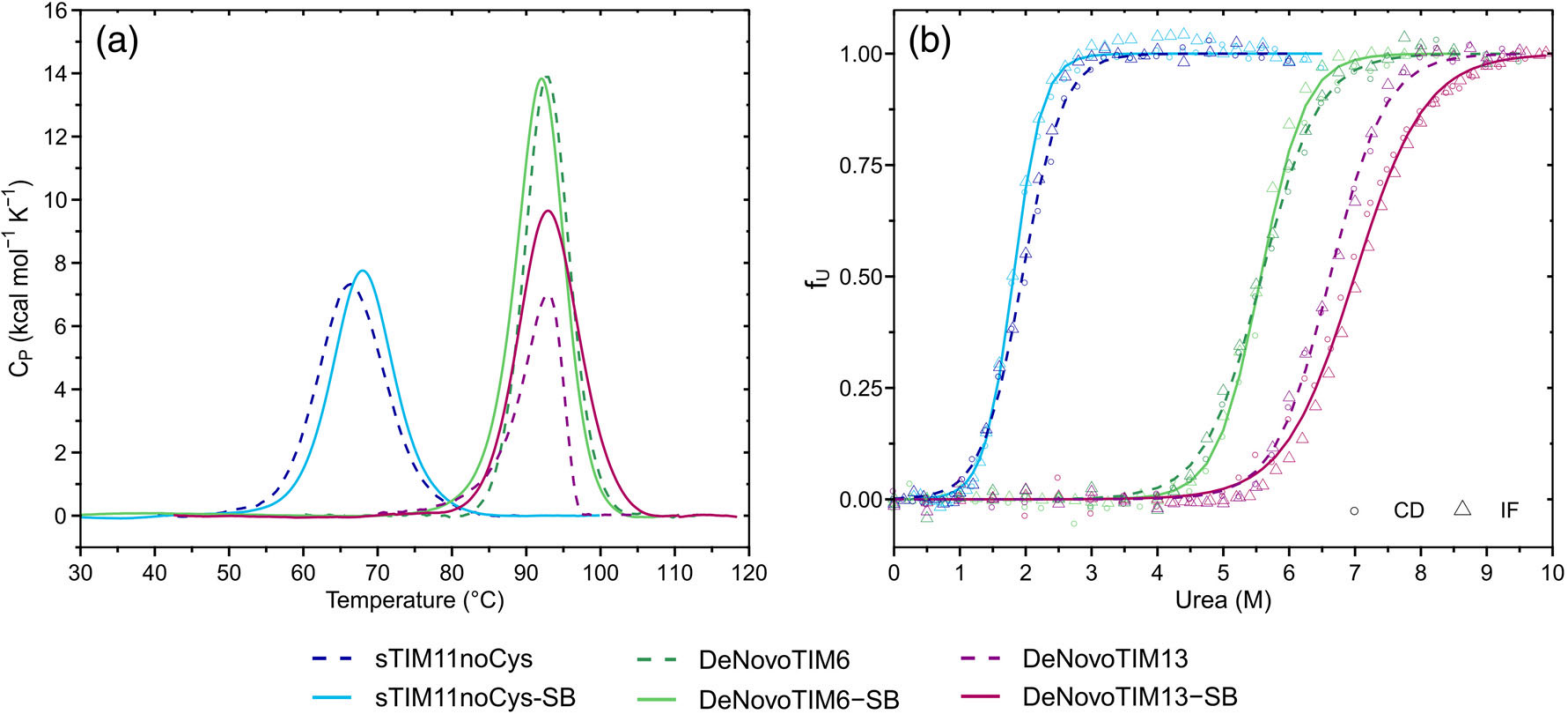
Folding stability of the salt bridge variants. (a) Thermal unfolding experiments followed by DSC. Endotherms were collected at 1.5°C min^−1^ and protein concentration of 1.0 mg ml^−1^. Dotted lines show the parental proteins and continuous lines the salt bridge cluster variants. (b) Chemical unfolding with urea at 25°C, circles representing CD data and triangles fluorescence data. Dotted and continuous lines represent the fitting of the data to a reversible two‐state model for the parental and salt bridge variants, respectively. Data from sTIM11noCys, DeNovoTIM6, and DeNovoTIM13 are reported in Reference 27. All experiments were collected in 10 mM sodium phosphate pH 8

Thermal stability was initially analyzed by CD (Figure S3). For sTIM11noCys‐SB, a melting temperature (*T*_m_) of about 64°C was determined. DeNovoTIM6‐SB and DeNovoTIM13‐SB both do not completely unfold in the accessible temperature range up to 95°C. Therefore, further analysis was performed by differential scanning calorimetry (DSC). For all three proteins no change of *T*_m_ was observed in comparison to the parental proteins (Table 1, Figure 2a), but with some changes in the enthalpy Δ*H*, mainly for DeNovoTIM13‐SB. Both, sTIM11noCys‐SB and DeNovoTIM6‐SB, show thermal unfolding reversibility (Figure S4a, S4c) and were fitted to a reversible two‐state model (Figure S4b, S4d).

**TABLE 1:** Thermodynamic parameters of salt bridge cluster variants in comparison with the parental proteins

*de novo* TIM barrel	Thermal unfolding (by DSC)						Chemical unfolding (by CD and IF)		
	*T*_m_ (°C)	Δ*H* (kcal mol^−1^)	Δ*H*_85°C_ (kcal mol^−1^)	Δ*C*_*P*_ (kcal mol^−1^ K^−1^)	Calorimetric criterion Δ*H*_vH_/Δ*H*	Global thermodynamic stability (kcal K mol^−1^)	Δ*G*_25°C_ (kcal mol^−1^)	*m* (kcal mol^−1^ M^−1^)	D_[1/2]_ (M)
sTIM11noCys^a^	65.6 ± 0.1	82 ± 1	128 ± 2	2.36 ± 0.08	0.99 ± 0.03	176	3.2 ± 0.2	2.03 ± 0.1	1.9
sTIM11noCys‐SB	67.4 ± 0.4	88 ± 2	123 ± 3	2.35 ± 0.09	1.00 ± 0.01	216	4.8 ± 0.1	2.66 ± 0.01	1.8
DeNovoTIM6^a^	92.3 ± 0.1	125 ± 2	108 ± 1	2.38 ± 0.06	1.03 ± 0.02	542	7.9 ± 0.2	1.51 ± 0.08	5.6
DeNovoTIM6‐SB	91.7 ± 0.1	128 ± 1	107 ± 1	2.09 ± 0.03	1.00 ± 0.03	684	9.8 ± 0.3	1.76 ± 0.06	5.6
DeNovoTIM13^a^	92.8 ± 0.4	47 ± 5	n.d, *E*_act_: 120 ± 3			n.d.	9.5 ± 0.2	1.54 ± 0.02	6.6
DeNovoTIM13‐SB	92.7 ± 0.3	100 ± 2	84 ± 1	1.48 ± 0.06	0.99 ± 0.02	596	8.2 ± 0.3	1.18 ± 0.03	6.9

*Note*: n.d., not determined due to irreversibility in the thermal unfolding. Instead, activation energy (*E*_act_) was calculated from an irreversible two‐state mechanism.

^a^ Parameters reported in Reference 27.

In contrast, DeNovoTIM13‐SB as well as DeNovoTIM13 show thermal‐unfolding irreversibility. Moreover, the behavior of DeNovoTIM13‐SB is remarkably different to that of DeNovoTIM13: the *T*_m_ in the salt‐bridge variant is only slightly dependent on the scan rate (Figure S5a) and the area recovered in the DSC reversibility test increased (Figure S4e), changing from 14% for DeNovoTIM13 to 72% for DeNovoTIM13‐SB. The lack of significant scan rate effects was demonstrated using a wide scan rate range from 0.5 to 3.0°C min^−1^ as previously suggested,^32^ confirming that the degree of recovery observed in DeNovoTIM13‐SB does not cause distortions in the baselines or thermal transition, which validate an equilibrium thermodynamics analysis (Figure S4f).

This combination of irreversibility and lack of scan rate dependence has been rarely reported. Typically, calorimetric irreversibility is caused by protein aggregation, but the lack of scan rate effect can be interpreted by assuming that the processes causing irreversibility only take place at very high temperatures where the protein is already completely unfolded.^33^ In addition to protein aggregation, there exists the possibility that swapped oligomers are formed in the unfolding state of DeNovoTIM13‐SB, which would be thermodynamically more similar to unfolded monomers rather than aggregates, therefore allowing a proper fitting to a reversible model. However, due to the high *T*_m_ of this protein, a more in‐depth analysis of the unfolded state with techniques such as CD and fluorescence spectroscopy, or SEC‐MALS is not possible.

Although the rule of thumb for thermal‐unfolding reversibility considers a recovered area higher than 85% for a reversible process, the thermodynamic behavior of DeNovoTIM13‐SB, that is, calorimetric irreversibility and no scan rate effects, allows to fit the endotherms to a reversible two‐state model as has been reported.^33^ In fact, when comparing the fitting for both the irreversible and reversible two‐state models (Figure S5b,c), the reversible model fits and explains the experimental data much better than the irreversible one. Also, the calorimetric criterion (Δ*H*_vH_/Δ*H*) is very close to 1, which is in agreement with a two‐state mechanism (Table 1). All these results confirm the suitability of this model to calculate the thermodynamic parameters for DeNovoTIM13‐SB.

**FIGURE 3:**
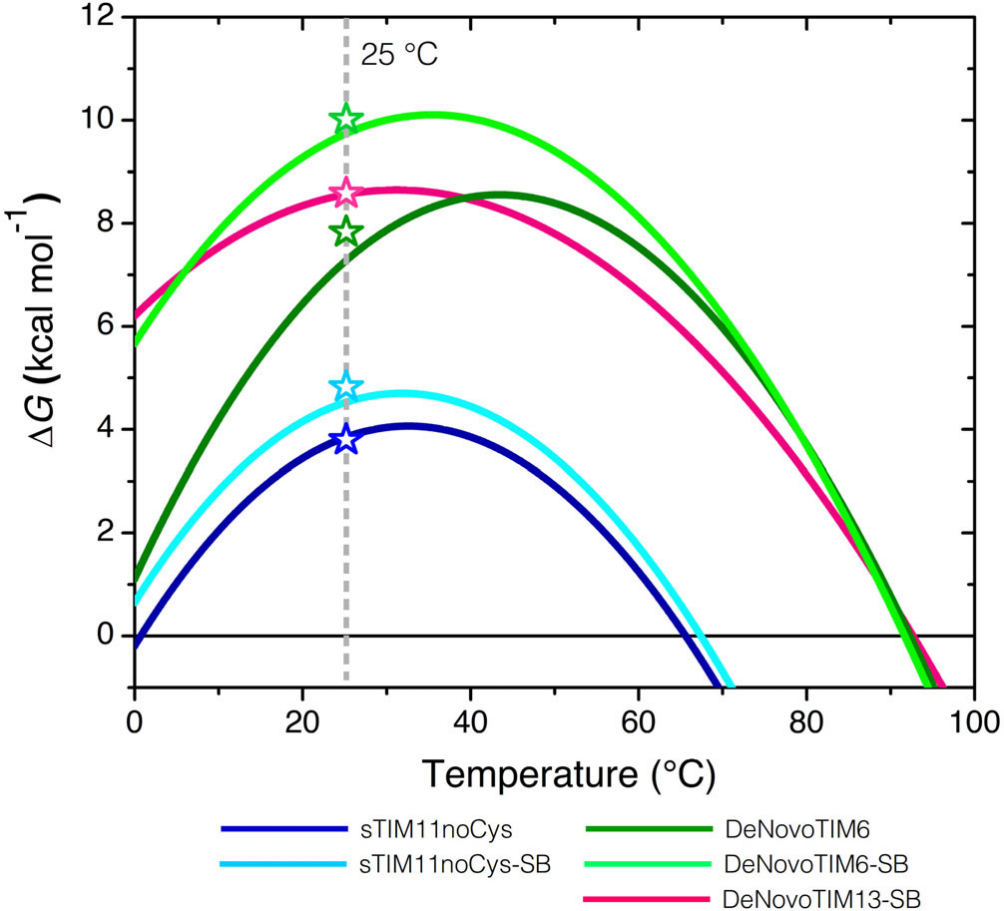
Stability curves of the salt bridge variants. Curves were constructed using the parameters from DSC experiments and the Gibbs–Helmholtz equation. Open symbols indicate the Δ*G* value at 25°C determined by chemical unfolding. Data from sTIM11noCys and DeNovoTIM6 are reported in Reference 27

Thermodynamic parameters determined for the salt bridge variants indicate no major changes when compared to the parental proteins (Table 1). The main difference is observed in the heat capacity change (Δ*C*_*P*_), which reshapes the stability curve without modifying the *T*_m_ (Figure 3) but increases the conformational stability at 25°C as discussed in the next section. Interestingly, no major changes in thermodynamic stability are observed regarding the *T*_m_. This stands in contrast to the assumption that salt bridges increase mostly the thermal stability of proteins as observed in thermophilic proteins.^34^, ^35^, ^36^, ^37^ Also, the web server for protein stabilization called *P*rotein *R*epair *O*ne *S*top *S*hop (PROSS) creates thermally stabilized protein by introducing salt bridges,^6^ though with the objective to influence the solvent exposure and to strengthen Coulomb interactions in the low‐dielectric protein core.^1^ The mutations that introduce salt bridges may have different effects on folding energies depending on the temperature;^1^, ^38^ this can only be answered if conformational stability would be analyzed at different temperatures. Here, upon introduction of the salt bridge cluster most of the differences are observed on the conformational stability at 25°C.

### 2.4 Salt bridge cluster variants have a higher conformational stability at 25°C

Changes in the conformational stability at 25°C (Δ*G*_25°C_) were studied by chemical unfolding with urea followed by CD and IF. All three proteins showed reversible and cooperative transitions. These fitted well to a two‐state model (N⇋U) (Figure 2b) with coincident Δ*G*_25°C_ values to those calculated from thermal unfolding experiments (Figure 3). Comparison of the salt bridge variants with the parental proteins exhibited different trends: In sTIM11noCys‐SB, the salt bridge cluster stabilized the protein by an increase of 1.6 kcal mol^−1^ in Δ*G*_25°C_, where the midpoint urea unfolding concentration (D_[1/2]_) stayed unchanged but the *m* value increased by 0.63 kcal mol^−1^ M^−1^ (Table 1), indicating an improved protein packing. Interestingly, the salt bridge cluster induces in sTIM11noCys‐SB a similar stability as observed for sTIM11, namely 4.8 kcal mol^−1^.^27^ sTIM11 contains two additional cysteines that are close in proximity but do not appear to form a disulfide bond. However, sTIM11 has a lower *m* value but an increased D_[1/2]_ compared to sTIM11noCys and sTIM11noCys‐SB. For DeNovoTIM6‐SB a similar trend is observed but with an even larger stabilizing effect of 1.9 kcal mol^−1^ resulting in a Δ*G*_25°C_ of 9.8 kcal mol^−1^ due to an increase of *m* value by 0.25 kcal mol^−1^ M^−1^. On the other hand, in DeNovoTIM13‐SB the salt bridge cluster seems to destabilize the protein slightly by −1.3 kcal mol^−1^ resulting in a reduced Δ*G*_25°C_ of 8.2 kcal mol^−1^. Also, the *m* value is decreased by −0.36 kcal mol^−1^ M^−1^ with a slight increase of D_[1/2]_ (Table 1).

This shows that despite a similar context of the basic protein topology, the stability contribution of the salt bridge residues is different. In sTIM11noCys‐SB and DeNovoTIM6‐SB the salt bridge cluster has a clear stabilizing effect. On the contrary, a destabilizing effect is observed in DeNovoTIM13‐SB, although both effects are modulated by changes in the *m* value. To analyze the thermodynamic contribution of the salt bridge network in these *de novo* TIM barrels in detail, other approaches as computing the electrostatic energies by *in silico* mutation to their hydrophobic isosteres^39^, ^40^, ^41^ or estimating the stability changes using a double‐mutant cycles^19^, ^41^, ^42^, ^43^, ^44^ could be used. However, due to the cluster nature of the salt bridges introduced here, the complexity of the analysis would complicate determining the contribution of each residue. Nonetheless, the stability changes in the salt bridge variants were correlated with the structural rearrangements that took place in the barrel when the salt bridge cluster was introduced.

### 2.5 *The mutations improve crystallization properties of the* de novo *TIM barrels*

**FIGURE 4:**
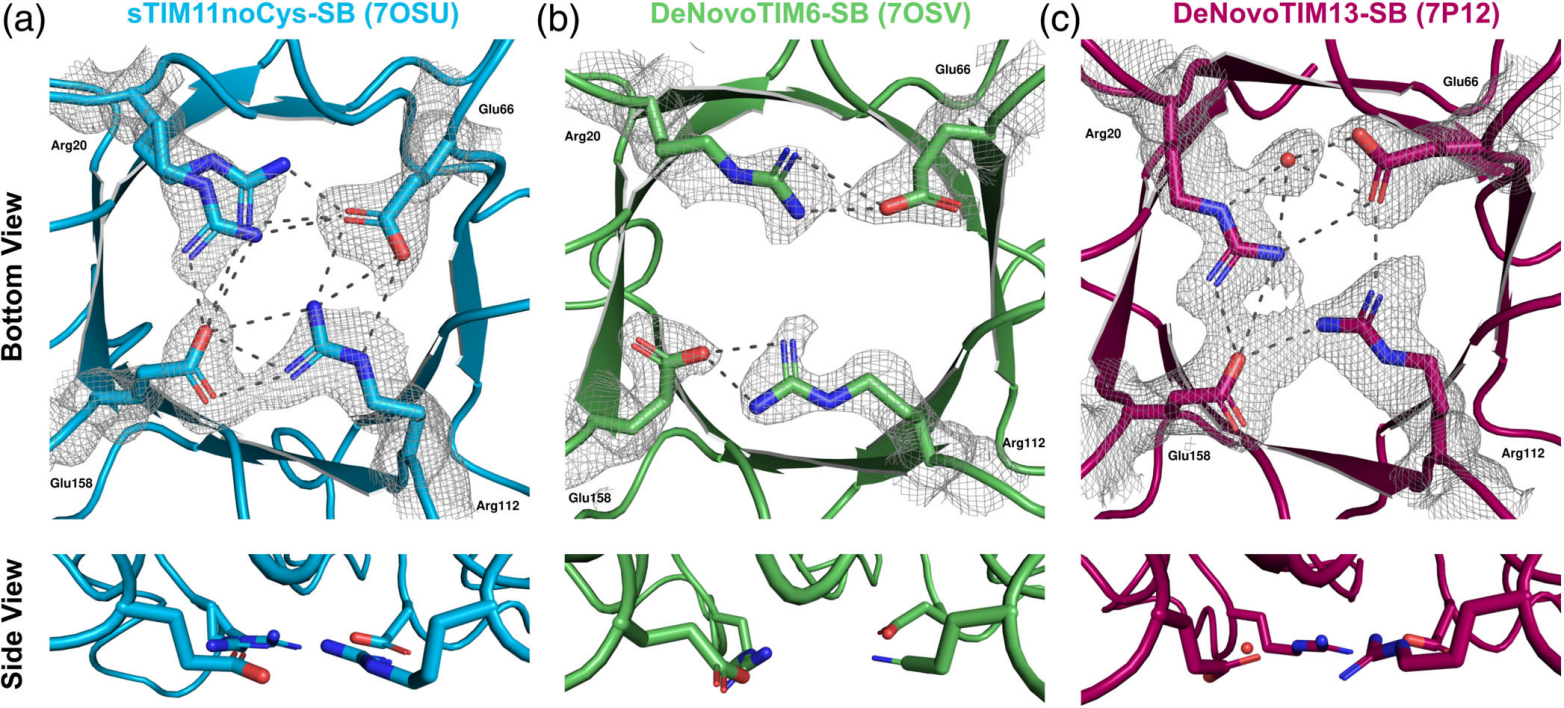
Structural conformations of the salt bridge interactions in the *de novo* TIM barrels. (a) sTIM11noCys‐SB (crystal form 1, PDB ID: 7OSU). (b) DeNovoTIM6‐SB (crystal form 1, PDB ID: 7OSV). (c) DeNovoTIM13‐SB (PDB ID: 7P12). In all panels, upper figures indicate the view from the bottom of the barrel with the salt bridge residues highlighted in sticks. 2Fo–Fc electron density maps contoured at 1σ are shown as a gray mesh for all the residues/water involved in the salt bridge cluster. Lower figures show the side view of the salt bridge interactions to analyze their planarity. Dotted lines indicate the salt bridge interactions between the mutated residues whose measures are reported in Table 2

In order to analyze the structural effects of the salt bridge mutations on the *de novo* TIM barrels, the three‐dimensional structures were solved by protein crystallography (Figures 4, S6, and Table S3). For all three parental proteins, the three‐dimensional structures were solved previously.^27^ For sTIM11noCys and DeNovoTIM13 crystallization was straightforward and structures were solved at high resolution. In contrast, crystallization and structure determination of DeNovoTIM6 was challenging. Despite a large number of crystals in screening and trials of several post crystallization treatments, only a low‐resolution structure could be solved. Based on this experience, we wanted to analyze and compare the crystallization properties of the salt bridge variants with those of the parental barrels.

Crystallization of all three salt bridge variants started with screening of several conditions. sTIM11noCys‐SB crystallized in the same space group as sTIM11noCys but the resolution was considerably improved from 1.88 to 1.37 and 1.51 Å (resolution for the two different crystal forms observed at different pH conditions, respectively).

Similar to its parental protein, DeNovoTIM6‐SB yielded a high number of crystals from screening with a hit rate of about 15% and crystallization conditions favoring ammonium sulfate as precipitant. Nevertheless, the crystals showed improved diffraction quality as is reflected in a decreased mosaicity, improved diffraction patterns and particularly in an improved resolution from 2.9 Å for DeNovoTIM6 to 1.66 and 2.22 Å (crystal form 1 and 2, respectively, crystallized in different conditions and space groups).

Also DeNovoTIM13‐SB crystallized in many conditions during screening. The best diffracting crystal was found in a condition similar to the one of DeNovoTIM13 but had a different space group. We observed an interesting anomaly resulting from the high symmetry of the *de novo* TIM barrels: initial data processing assumed the space group I4, but in the associated unit cell dimensions only a quarter of the TIM barrel could be fitted (Figure S7a). Molecular replacement using only a quarter of DeNovoTIM13 yielded good scores, and symmetry operations resulted in a nicely reconstructed TIM barrel using the corresponding symmetry operations with the center of the protein located in the vertex of the unit cell (Figure S7b). Nevertheless, for structure determination, the dataset had to be processed using space group P1, which produced a unit cell with a volume large enough for a complete TIM barrel in the asymmetric unit. To achieve higher completeness and low radiation damage in the P1 space group, datasets from two crystals from the same condition were merged. Despite this interesting observation, it did not affect the diffraction quality as the structures of DeNovoTIM13 and DeNovoTIM13‐SB have similar resolutions of 1.64 and 1.69 Å, respectively.

Since DeNovoTIM6‐SB and DeNovoTIM13‐SB show a small dimer population during protein purification and SEC‐MALS measurements, a possible dimer generation in the crystal structures was analyzed using symmetry operators and noncrystallographic symmetry. However, in none of the cases the formation of the oligomer could be recapitulated that would hint at the structural properties of DeNovoTIM6‐SB and DeNovoTIM13‐SB dimers observed in solution. For DeNovoTIM6‐SB, even though crystallization was set up only with the monomeric protein fraction, one of the crystal structures (PDB ID: 7OT8) shows two molecules in the asymmetric unit; nevertheless, no tight interface interactions are observed in this crystal form.

These observations of improved crystallization can be correlated with the conformational stability of the proteins: sTIM11noCys‐SB and DeNovoTIM6‐SB both have an increased stability that in both cases is mediated by the *m* value, which is connected to the protein packing. These effects on the crystallization behavior and the data quality additionally raise interest in the exact geometry and how it might influence the protein fold. Therefore, the solved structures were analyzed in detail regarding the geometry of the cluster residues, and additional changes of the TIM barrel fold were investigated.

### 2.6 *The salt bridge residues arrange with different geometries on the* de novo *TIM barrels*

Several previous studies have pointed out that the contribution of a salt bridge to stability is connected with its geometry, that is, distances and angles.^10^, ^42^, ^45^, ^46^ A comprehensive overview of possible geometric orientations of salt bridges and a statistical analysis of their frequency is given by Donald et al.^10^ Analysis of the formed geometries of these residues in the solved crystal structures showed a varying behavior depending on the structural context of the *de novo* TIM barrel where the residues were introduced (Table 2).

**TABLE 2:** Salt bridge geometries of the *de novo* TIM barrels analyzed in this work

No	Residue 1	Residue 2	Distance (Å)	Angle (°)
sTIM11noCys‐SB (7OSU)				
1	Arg20A‐Nη1	Glu66‐Oε1	3.3	164.0
2	Arg20A‐Nη2	Glu66‐Oε1	2.7	
3	Arg20A‐Nη1	Glu158‐Oε2	3.8	108.0
4	Arg20B‐Nη2	Glu158‐Oε2	3.1	101.4
5	Arg20B‐Nη2	Glu158‐Oε2	3.6	158.8
6	Arg20B‐Nη1	Glu158‐Oε2	2.6	
7	Arg112‐Nη2	Glu66‐Oε1	3.2	95.9
8	Arg112‐Nη2	Glu66‐Oε2	3.4	65.8
9	Arg112‐Nε	Glu66‐Oε2	3.6	
10	Arg112‐Nη1	Glu158‐Oε1	2.7	137.8
11	Arg112‐Nη1	Glu158‐Oε2	3.2	170.0
12	Arg112‐Nη2	Glu158‐Oε2	3.7	
DeNovoTIM6‐SB (7OT8)				
1	Arg20‐Nη1	Glu66‐Oε2	3.5	163.2
2	Arg20‐Nη2	Glu66‐Oε2	2.9	
3	Arg112‐Nη1	Glu158‐Oε2	3.0	158.2
4	Arg112‐Nη2	Glu158‐Oε2	2.8	
DeNovoTIM13‐SB (7P12)				
1	Arg20‐Nε	H2O29	2.7	52.3
2	Arg20‐Nη2	H2O29	3.1	
3	Arg20‐Nη2	Glu66‐Oε1	3.7	93.2
4	Arg20‐Nη1	Glu158‐Oε2	2.7	157.3
5	Arg20‐Nη2	Glu158‐Oε2	3.7	
6	H2O29	Glu66‐Oε2	2.6	107.3
7	H2O29	Glu66‐Oε1	3.1	81.1
8	Arg112‐Nη1	Glu66‐Oε1	3.1	134.6
9	Arg112‐Nη2	Glu158‐Oε2	3.0	110.0

The solved crystal structures of the salt bridge variants did not show deviations in their TIM‐barrel topology. Interestingly, in all three proteins different salt bridge geometries are observed. In sTIM11noCys‐SB, a highly ordered salt bridge cluster is formed quite similar to the intended geometry (Figure 4a). Arg20 shows two alternative conformations, providing two different salt bridge sets. One conformation (Arg20‐A) forms two salt bridge interactions with Glu66 and one with Glu158. The alternative conformation Arg20‐B similarly forms two salt bridges with Glu158 and one with Glu66. Arg112 forms a highly coordinated side‐on salt bridge with Glu66 and additionally interacts with Glu158 in an end‐on configuration, in both cases via three interactions (Table 2). These two configurations are expected to be lowest‐energy states based on quantum mechanics calculations.^10^ A single energetically favorable salt bridge must have a good balance between the unfavorable entropic cost and favorable coulombic interactions. In the case of a salt bridge cluster formation as observed in sTIM11noCys‐SB, the entropic cost should be lower than for a single one, as one side chain is already reduced in its degrees of freedom.^42^

The structural analysis of the DeNovoTIM6‐SB salt bridge residues revealed a different configuration. In contrast to sTIM11noCys‐SB, no cluster is formed between the four residues and only two independent salt bridge pairs are observed between Arg20‐Glu66 and Arg112‐Glu158. In both cases, two interactions between arginine and glutamate in a monodentate backside configuration are made (Figure 4b). This reduced number of interactions in comparison to sTIM11noCys‐SB does not correlate directly with the changes in stability for these two proteins, where for sTIM11noCys‐SB the stability is increased by 1.6 kcal mol^−1^ compared to sTIM11noCys, and DeNovoTIM6‐SB increased by 1.9 kcal mol^−1^ compared to DeNovoTIM6‐SB (Table 1).

For DeNovoTIM13‐SB, again a different configuration of the introduced network is observed in this case involving a water molecule (Figure 4c). A bidentate water‐mediated salt bridge is formed between Arg20 and Glu66 additionally to a monodentate direct salt bridge. In addition, Arg20 interacts with Glu158, which also interacts via a single salt bridge with Arg112. Closing the network another single salt bridge is formed between Glu66 and Arg112 (Table 2). The reduction on the conformational stability of DeNovoTIM13‐SB compared to DeNovoTIM13 would indicate possible negative influences of the salt bridge cluster on the topology. Therefore, the highly coordinated salt bridge network observed in the crystal structure indicates that other structural rearrangements take place in different regions of the barrel. Considering the approach followed to design DeNovoTIM13 that aimed on the improvement of hydrophobic clusters,^27^ changes in these clusters were analyzed in DeNovoTIM13‐SB as a possible cause for the reduction in stability. In fact, we observed that DeNovoTIM13‐SB exhibits a reduction of 409 Å^2^ in the total area of the hydrophobic clusters in comparison to DeNovoTIM13 (5,972 vs. 6,381 Å^2^, respectively). Since epistatic effects play an important role in the stabilization of DeNovoTIMs, a likely reason why DeNovoTIM13‐SB reduces in stability is that the introduction of the salt bridge cluster promotes rearrangements that modify the hydrophobic clusters.

Another suggested factor for the stability of a salt bridge is its planarity, meaning a higher planarity implies a better coordination of the charged residues resulting in a higher contribution to the stability.^10^, ^47^ Analysis of the three different salt bridge clusters shows highest planarity for sTIM11noCys‐SB (Figure 4a bottom), followed by DeNovoTIM13‐SB with a slight distortion of Arg112 (Figure 4C bottom) and lowest planarity in DeNovoTIM6‐SB, which is most likely due to the absence of a well‐formed cluster geometry (Figure 4b bottom). In addition to the absence of a full cluster in DeNovoTIM6‐SB and the therefore low overall planarity between the four residues, also the interacting residue pairs have a low planarity indicating a poor coordination of the separated salt bridges.

Possible influence of the crystal packing on the salt bridge geometries were analyzed by comparing the Matthews coefficient (V_m_) and solvent content of the different variants (Table S3). Crystals of sTIM11noCys‐SB and DeNovoTIM6‐SB have a similar V_m_ of about 1.9 leading to a solvent content of 36% and 37%, respectively. DeNovoTIM13‐SB on the other hand has a higher V_m_ of 2.25 with a solvent content of 45%. We deduce that the better coordinated geometry of sTIM11noCys is not induced by a tighter crystal packing, as DeNovoTIM6‐SB has a similar solvent content.

Finally, since the design and prediction of salt bridge networks and their corresponding changes in stability are open challenges, we tested if the determined changes in stability observed in the salt bridge variants could be predicted by Rosetta scoring.

### 2.7 Rosetta recapitulates the changes in stability but not the salt bridge geometries

Prediction and modeling of salt bridges is challenging due to the importance of a well‐formed geometry of the involved residues. As we did not perform any preceding modeling of the introduced salt bridge cluster but based it on similar clusters observed in the natural TIM barrel HisF,^28^, ^29^ we were interested if the introduced electrostatic interactions could be accurately modeled and scored according to our experimental data. Therefore, the mutated residues were introduced into the parental protein structures using Rosetta Remodel followed by relaxation of the models.^48^ Analysis of the created models shows that a complete network is rarely built and also does not score best. Nevertheless, most of the decoys show partially formed networks with one arginine frequently pointing out. Obviously, generating the geometry observed in NovoTIM13‐SB involving a water molecule is not possible with this design approach, as no water molecules are included. Still, a more optimized approach might have led to a better performance.

To analyze the influence of a well‐placed salt bridge network on the Rosetta score, the crystal structures of the salt bridge variants as well as the parental proteins were scored using the most recent and default scoring function *ref2015* in Rosetta.^49^ First, all structures were idealized using rosetta.relax with constrained backbone and sidechain geometries to the starting structure.^50^ Due to varying residue numbers of the structures, the Rosetta scores were normalized to the total number of residues. Comparison of the scores of each parental protein with its corresponding salt bridge variant showed a decrease of the total score for sTIM11noCys‐SB and DeNovoTIM6‐SB by −1.24 and −1.37 Rosetta energy units (REU), respectively. However, DeNovoTIM6 scores really low with a total of −0.12 REU per residue due to the low resolution of the structure and the associated poor model quality including missing side chains. In contrast, DeNovoTIM13‐SB shows an increase of the total score by 0.23 REU compared to DeNovoTIM13 (Table S4).

Comparing the experimentally determined stability for these proteins with the corresponding scores shows that Rosetta is predicting a similar trend for all proteins regarding their stability. For sTIM11noCys and DeNovoTIM6 the score indicates an increase in stability with the addition of the salt bridge cluster, which was verified in the unfolding experiments. Also, the scores of DeNovoTIM13 and DeNovoTIM13‐SB are in agreement with the experiments, showing that in this context the salt bridge cluster does not have a positive effect on the stability.

Collectively, using a minimalistic design approach with Rosetta it was not possible to generate models similar to the final solved structures, which might be improved with the introduction of specific constraints and scores. Nevertheless, scoring of the native salt bridge clusters reveals clearly that the most recent scoring function perceives the cluster and considers it positively.

## 3 CONCLUSIONS

The engineering, design and prediction of the stabilizing effect of salt bridges in proteins is a challenging task due to the high interdependency of various factors. Despite the necessity for an optimal geometry, a stabilizing effect is only achieved with the compensation of the desolvation penalty by the bridging energy and other interactions caused by conformational changes.

Here we studied the effects on structure and stability by the introduction of a salt bridge cluster into three different *de novo* TIM barrels. In contrast to findings of previous studies, which correlated the increased thermostability of many proteins with an increased number of salt bridges, our analysis showed no influence on the *T*_*m*_ values for all three proteins. In contrast, analysis of the conformational stability at 25°C revealed different stabilizing effects: in sTIM11noCys‐SB and DeNovoTIM6‐SB, a clear stabilization by 1.6 and 1.9 kcal mol^−1^ was observed, respectively. In contrast, DeNovoTIM13‐SB is destabilized through the introduced mutations by −1.3 kcal mol^−1^. Nevertheless, also in DeNovoTIM13‐SB the salt bridge cluster has a positive effect in the reduction of aggregation‐propensity most likely by the change from an irreversible thermal unfolding process to a reversible one. Our results highlight the complexity of salt bridges in proteins: despite the high identity in sequence and structure of all three proteins, the similar salt bridge clusters have clearly different stabilizing effects.

In addition, we observed improvements on the crystallization properties of the *de novo* TIM barrels in comparison with the parental proteins. The structural analysis revealed highly diverse geometries for all three proteins, ranging from the absence of a cluster geometry and the formation of single salt bridges, via a water mediated cluster arrangement to a highly coordinated cluster network. Interestingly, the network geometry does not correlate with the corresponding stability. For instance, the crystal structure of DeNovoTIM6‐SB revealed only the presence of two single salt bridges but the highest stabilizing effect. Due to these diverse influences of the salt bridge cluster on highly similar *de novo* TIM barrels, the influence of salt bridges could be studied in more detail to partition the stabilizing and destabilizing components under the same topology.

Most *de novo* protein design approaches lack intensive design of salt bridges and especially clusters, despite their proven importance for stability and function. Our analysis of the salt bridge TIM barrel variants indicate that Rosetta is able to predict influences on the stability with the right tendency. Nevertheless, the modeling of an accurate cluster geometry is still challenging in a fully automated approach. The engineering and design of salt bridge clusters in different natural and *de novo* proteins would benefit from an improved understanding of salt bridges.

## 4 MATERIALS AND METHODS

### 4.1 Biochemicals

All reagents were analytical grade from Sigma‐Aldrich or Carl Roth, except when indicated. All solutions were prepared with double‐distilled water.

### 4.2 Cloning, overexpression, and protein purification

All genes were synthesized and cloned into pET21b(+) vector by BioCat. *Escherichia coli*, BL21(DE3) (Novagen) were transformed with plasmids and used to inoculate LB precultures supplemented with ampicillin (100 μg ml^−1^) which were grown at 37°C and 180 rpm overnight. Overexpression was performed in 1 L Terrific Broth (TB) cultures inoculated on OD_600_ 0.08 and then grown at 37°C. At an OD_600_ of 0.8–1 overexpression was induced with 1 mM isopropyl‐d‐1‐thiogalactopyranoside and growth performed at 30°C for 4.5 hr. Afterward, cells were harvested by centrifugation (Beckmann Avanti JLA‐8.1000, 15 min, 5,000*g*, 4°C) and pellets resuspended in 5 ml per gram pellet with buffer A: 35 mM sodium phosphate, 150 mM NaCl, 35 mM Imidazole, pH 8 (supplemented with 100 μl protease inhibitor [Mix‐HP, Serva] per 10 ml lysate). Cells were lysed by sonication (Branson Ultrasonics) (output 4, duty cycle 40%, 2 times 2 min) and then centrifuged (Beckmann Avanti JA‐25.50, 1 hr, 18,000 rpm, 4°C). The lysate was filtered with a 0.22 μm filter (Merck Millipore) and loaded onto a HisTrap HP column (5 ml, Cytiva) equilibrated with buffer A and coupled to an Äkta system (GE Healthcare Life Sciences). Unbound proteins were washed out with 20 column volumes (CVs) of buffer A. Elution of bound protein was performed with a linear gradient over 20 CV from 35 to 300 mM Imidazole using buffer B (35 mM sodium phosphate, 150 mM NaCl, 500 mM Imidazole, pH 8) followed by a step to 500 mM Imidazole for 5 CV. The peak fractions were pooled, concentrated and loaded onto a HiLoad 16/600 Superdex 500 preparative grade column (GE Healthcare Life Sciences) connected to an Äkta System. Elution was performed with 1 CV buffer C (35 mM sodium phosphate, 150 mM NaCl, pH 8) and the monomeric peak fractions were pooled and stored at room temperature or 4°C for use in subsequent experiments. For some subsequent experiments the proteins were dialyzed into buffer D (10 mM sodium phosphate, pH 8).

### 4.3 Analytical size exclusion chromatography‐multi angle light scattering (SEC‐MALS)

SEC‐MALS measurements were performed using a Superdex 75 Increase 10/300 GL column (GE Healthcare Life Sciences) connected to an Äkta Pure System, and coupled to a miniDAWN multi‐angle light scattering detector and an Optilab refractometer (WyattTechnology). All experiments were performed in buffer C with 0.02% sodium azide at room temperature and a flow rate of 0.8 ml min^−1^, using a protein concentration of 1 and 5 mg ml^−1^. Data collection and analysis were performed with the ASTRA 7.3.2 software (Wyatt Technology). To check for reproducibility during the SEC‐MALS runs, BSA standard sample at 2 mg ml^−1^ was measured at the beginning and end of each measurement day, obtaining identical results.

### 4.4 Far‐UV CD

CD spectra were collected in buffer D with a Jasco J‐710 using a Peltier device to control the temperature (PTC‐348 WI). Far‐UV CD spectra were measured with a protein concentration of 0.2 mg ml^−1^ in the wavelength range 195–260 nm at 25°C with a 1 nm bandwidth in a 2 mm cuvette. Spectra of thermally unfolded states were collected at 95°C. Data were normalized by subtraction of buffer spectra and then converted to mean residue molar ellipticity using: [*θ*_MRE_] = (*M*⋯*θ*)/(10⋯*d*⋯*c*) and *M* = (*MW*/*n* − 1), where *M* is the mean residue weight, *MW* is the molecular weight in Da, *n* is the number of residues in the protein, *θ* is the collected ellipticity in mdeg, *d* is the path length in mm, and *c* is the protein concentration in mg ml^−1^. Far‐UV spectra were deconvoluted with CDNN.^51^

### 4.5 Intrinsic fluorescence

Intrinsic fluorescence (IF) spectra were collected in buffer D with a protein concentration of 0.2 mg ml^−1^ using a Jasco FP‐6500 spectrofluorometer and a Peltier device to control the temperature (Julabo MB). Fluorescence was excited at a wavelength of 295 nm and emission was measured in the wavelength range 310–450 nm with a bandwidth of 1 nm. Spectra of unfolded protein were measured at a Urea concentration capable of unfolding the protein. The spectral center of mass was calculated using: $\mathrm{SCM}=\sum \lambda \frac{I_{\lambda }}{\sum }I_{\lambda }$.

### 4.6 Thermal unfolding followed by CD

Thermal unfolding was followed by CD at a protein concentration of 0.2 mg ml^−1^ in buffer D in a 2 mm cuvette. The unfolding was followed in the temperature range 20–95°C at 222 nm with a heating rate of 1.5°C min^−1^. Spectra were normalized to the fraction of unfolded molecules (*f*_*u*_) by:

(1) $$f_{u}=\frac{y_{\mathrm{obs}}-\left(y_{N}+m_{n}T\right)}{\left(y_{u}+m_{u}T\right)-\left(y_{N}+m_{N}T\right)}$$

with *y*_obs_ the observed CD signal at a given temperature, and (*y*_*N*_ + *m*_*N*_ *T*) and (*y*_*u*_ + *m*_*U*_ *T*) the linear fitting equations of the native and unfolded regions, respectively.

### 4.7 Thermal unfolding followed by DSC

Temperature‐induced unfolding experiments by DSC were collected in a VP‐Capillary DSC (Malvern Panalytical). Samples were assayed at 1.5°C min^−1^ and protein concentration of 1, 2, 3, and 4 mg ml^−1^ in buffer D, after exhaustive dialysis and buffer degassing. In all cases, proper equilibration was performed by running at least two buffer–buffer scans before sample‐buffer experiments. The last buffer–buffer scan was subtracted from each protein‐buffer scan to perform all thermodynamic analysis. Reversibility was determined by collecting a second endotherm after the first one was collected. For DeNovoTIM13‐SB, endotherms were also collected at 1 mg mL^−1^ and varying scan rate from 1 to 3°C min^−1^. DSC scans were fitted to a two‐state reversible model (Equation 2):

(2) $$C_{P}\left(T\right)=B_{0}+B_{1}T+f\left(T\right)\text{Δ}C_{P}+\frac{\text{Δ}H\left(T\right)}{RT_{m}^{2}}\left[\frac{1-f\left(T\right)}{1-n+\frac{n}{f\left(T\right)}}\right]$$

where *B*_0_ and *B*_1_ are pre‐ and post‐transition constants, *n* is the number of subunits in the native protein sample (monomer for all the proteins in this work) and *f*(*T*) is the protein fraction in the folded monomeric state, yielding the parameters Δ*H*, Δ*C*_*P*_, and *T*_m_. To test the accuracy of the fitting, the DeNovoTIM3‐SB endotherm at 1 mg ml^−1^ was also fitted to an irreversible two‐state model as indicated in Reference 27. Origin v.7.0 (OriginLab Corporation) with MicroCal software was used for data analysis.

Stability curves were constructed using DSC parameters and the Gibbs–Helmholtz equation:^52^

(3) $$\text{Δ}G\left(T\right)=\text{Δ}H\left(1-\frac{T}{T_{m}}\right)-\text{Δ}C_{P}\left(T_{m}-T+\mathrm{Tln}\left(\frac{T}{T_{m}}\right)\right)$$

### 4.8 Chemical‐induced unfolding followed by CD and IF

For chemical‐induced unfolding experiments protein concentration was 0.2 mg ml^−1^ in buffer D. Initially, the equilibrium time for chemical unfolding was determined by incubation of samples at different urea concentrations (0–9 M). CD and IF spectra were recorded at different incubation times and 2 days are sufficient for all analyzed proteins to reach equilibrium. Chemical unfolding experiments were carried out by incubation of samples with increasing urea concentration for 2 days at 25°C. For all urea concentrations the CD signal at 222 nm was measured for 2 min and IF spectra were recorded as aforementioned at 25°C. IF data were processed considering the intensity ratio at the wavelength of the maximum of the unfolded spectrum (*I*_*λu*_) and at the wavelength of the maximum of the native spectrum (*I*_*λn*_) at every urea concentration ($r_{\lambda }=\frac{I_{\lambda^{u}}}{I_{\lambda^{n}}}$). IF and CD data at every urea concentration were normalized to the fraction of unfolded protein using Equation (4), where *y*_obs_ is the experimentally observed CD signal or the calculated ratio of IF data at a given concentration, and (*y*_*N*_ + *m*_*N*_[urea]) and (*y*_*U*_ + *m*_*U*_[urea]) are the linear fitting equations of the native and unfolded regions, respectively.

(4) $$f_{U}=\frac{y_{\mathrm{obs}}-\left(y_{N}+m_{N}\left[\text{urea}\right]\right)}{\left(y_{U}+m_{U}\left[\text{urea}\right]\right)-\left(y_{N}+m_{N}\left[\text{urea}\right]\right)}$$

Determination of the unfolding free energy Δ*G* ^H2O^ was performed by fitting of the data to a two‐state model (N⇌D) using the Santoro and Bolen equation (Equation 5)^53^:

(5) $$f_{U}=\frac{\left(y_{N}+m_{N}\left[\text{urea}\right]\right)+\left(y_{U}+m_{U}\left[\text{urea}\right]\right)*\exp\left(\frac{-{\text{Δ}G}^{H_{2}O}-m\left[\text{urea}\right]}{\mathrm{RT}}\right)}{1+\exp\left(\frac{-\text{Δ}G^{H_{2}O}-m\left[\text{urea}\right]}{\mathrm{RT}}\right)}$$

where *m* is Δ*G*/[urea], a parameter related with the dependence of free energy on denaturant concentration and commonly associated with unfolding cooperativity, proportional to the surface area of protein exposed to solvent upon unfolding;^54^, ^55^ *T* is the temperature of the experiment (298.15 K), and R the universal gas constant (0.001987 kcal mol^−1^ K^−1^). In addition, the denaturant concentration at the midpoint of the unfolding curve, D_[1/2]_ reported in Table 1, is equivalent to D_[1/2]_ = Δ*G* ^H2O^/*m*.^55^ Data analysis and fitting was conducted with R^56^ and graphs were created with package ggplot2.^57^

### 4.9 Crystallization and structure determination

For crystallization all proteins were in buffer C. Initial screening was performed with the sitting‐drop vapor diffusion method using JCSG Core I‐IV, Classics I‐II, PEGs I‐II (Qiagen) in 96‐well Intelli plates (Art Robbins Instruments) using a nano dispensing crystallization robot Phoenix (Art Robbins Instruments). Crystallization drops with a volume of 0.8 μl were prepared with different ratios of mother liquid and protein (1:1, 1:2, 2:1). Screening plates were stored at 20°C in the hotel‐based Rock Image RI 182 (Formulatrix). Crystallization hits were optimized using sitting and hanging drop vapor diffusion in MRC Maxi 48‐well plates and VDXm 24‐well plates, respectively, with a crystallization drop size of 2 μl. Initial screening was performed with 10 mg ml^−1^ (sTIM11noCys‐SB), 5.9, 8.6, 9, and 12 mg ml^−1^ (DeNovoTIM6‐SB) and 8.25 mg ml^−1^ (DeNovoTIM13‐SB).

In the following conditions good diffracting crystals were found: sTIM11noCys‐SB (crystal form 1): 50% PEG 200, 0.2 M NaCl, 0.1 M phosphate citrate, pH 5, drop ratio 2:1 (protein: mother liquid); sTIM11noCys‐SB (crystal form 2): 34% PEG 200, 0.1 M NaCl, 0.1 M Tris pH 7.78, 0.2 M lithium sulfate, drop ratio 1:1 (protein: mother liquid); both conditions with a protein concentration of 10 mg ml^−1^. DeNovoTIM6‐SB (crystal form 1): 0.2 M ammonium sulfate, 0.2 M sodium acetate, pH 4.6, 28% PEG 4000 with a drop ratio of 1:1; DeNovoTIM6‐SB (crystal form 2): 0.2 M ammonium sulfate, 0.2 M sodium acetate, pH 4.3, 31% PEG 4000 with a drop ratio of 1:1; both conditions with a protein concentration of 8.6 mg ml^−1^. DeNovoTIM13‐SB: 0.17 M sodium acetate trihydrate, 0.085 M Tris, pH 8.9, 23% PEG 4000, 15% glycerol with a protein concentration of 8.4 mg ml^−1^ and a drop ratio of 1:1.

For DeNovoTIM6‐SB diffraction data were collected at 100 K at the Swiss Light Source at the Paul Scherrer Institute in Villigen (Switzerland) (PXI beamline) using a wavelength of 1.00 and a EIGER 16 M X Detector (Dectris).^58^ For sTIM11noCys‐SB and DeNovoTIM13‐SB diffraction data were collected at 100 K at the Berlin Electron Storage Ring Society for Synchrotron Radiation beamline 14.1 and 14.2 (BESSY 14.1 and 14.2) operated by Helmholtz‐Zentrum Berlin using a wavelength of 0.9184 Å and a PILATUS3 S 6 M or PILATUS3S 2 M detector, respectively.^59^

The datasets were processed with the X‐ray detector software (XDS) using XDSAPP v3.0^60^, ^61^ or command line. For DeNovoTIM13‐SB two datasets of two different crystals from the exact same condition were merged using XSCALE to achieve a higher completeness in space group P1. Molecular replacement was performed with PHASER in the PHENIX software suite v.1.19.2^62^ using sTIM11noCys (PDB ID: 6YQY) as a starting model for sTIM11noCys‐SB and DeNovoTIM13 (PDB ID: 6YQX) for DeNovoTIM6‐SB and DeNovoTIM13‐SB. Structure refinement was performed with phenix.refine^63^ and iterative manual model improvement by rebuilding in COOT v.0.9.^64^ Coordinates and structure factors were deposited in the PDB database https://www.rcsb.org/ ^65^ with the accession codes: 7OSU (sTIM11noCys‐SB, crystal form 1), 7OT7 (sTIM11noCys‐SB, crystal form 2), 7OSV (DeNovoTIM6‐SB, crystal form 1), 7OT8 (DeNovoTIM6‐SB, crystal form 2), and 7P12 (DeNovoTIM13‐SB). Secondary structure composition of crystal structures was calculated using the STRIDE Web Interface,^66^ (http://webclu.bio.wzw.tum.de/cgi-bin/stride/stridecgi.py). The figures were created using PyMOL Molecular Graphics System v.4.6.0 (Schrodinger, LLC).

### 4.10 Geometric analysis of the salt bridge cluster

In detail analysis of the salt bridge cluster geometries was performed in PyMol (Schrodinger, LLC). Distances reported were calculated by measuring the distance between the corresponding heavy atoms. Measurements of the angles for a certain salt bridge were performed as previously suggested^10^ measuring the angle between ∢(Arg‐N^ε^, Arg‐C^ζ^, Glu‐O^ε^). In case a water molecule was involved in the salt bridge, either ∢(Arg‐N^ε^, Arg‐C^ζ^, H_2_O) or ∢(Glu‐C^δ^, Glu‐O^ε^, H_2_O) were determined depending on the involved residue type.

### 4.11 Rosetta calculations

Crystal structures of the parental proteins (sTIM11noCys—6YQY, DeNovoTIM6—6YQX, DeNovoTIM13—6Z2I) as well as of the salt bridge cluster variants (sTIM11noCys‐SB—7OSU, DeNovoTIM6‐SB—7OSV, DeNovoTIM13‐SB—7P12) were scored with Rosetta using the *ref2015* scoring function.^49^ The PDB structures were initially cleaned with the clean_pdb.py script from Rosetta tools followed by a relax with constraining the structure, backbone and side chains, to input coordinates.^50^

Rosetta models were created by mutation of the salt bridge cluster residues using Remodel.^48^ As a starting model, for sTIM11noCys‐SB and DeNovoTIM13‐SB the parental protein structures were used, whereas for NovoTIM6 the previously created Rosetta model was used.^27^ Subsequently, the models and crystal structures of the parental as well as of the salt bridge cluster variants were relaxed via an iterative approach until no further decrease of the score was observed.

## CONFLICT OF INTEREST

The authors declare no potential conflict of interest.

## AUTHOR CONTRIBUTIONS

**Sina Kordes:** Conceptualization (equal); data curation (equal); formal analysis (equal); investigation (equal); methodology (equal); software (equal); supervision (equal); validation (equal); visualization (equal); writing – original draft (equal); writing – review and editing (equal). **Sergio Romero‐Romero:** Conceptualization (equal); data curation (equal); formal analysis (equal); investigation (equal); methodology (equal); supervision (equal); validation (equal); visualization (equal); writing – original draft (equal); writing – review and editing (equal). **Leonie Lutz:** Formal analysis (equal); investigation (equal); methodology (equal); writing – review and editing (equal). **Birte Höcker:** Conceptualization (equal); funding acquisition (equal); project administration (equal); resources (equal); supervision (equal); writing – original draft (equal); writing – review and editing (equal).

## Supporting information


**Appendix** **S1**: Supporting Information

## Data Availability

All data to support the conclusions of this manuscript are included in the main text and supporting information. Coordinates and structure factors have been deposited to the Protein Data Bank (PDB) with accession codes: 7OSU (sTIM11noCys‐SB, crystal form 1), 7OT7 (sTIM11noCys‐SB, crystal form 2), 7OSV (DeNovoTIM6‐SB, crystal form 1), 7OT8 (DeNovoTIM6‐SB, crystal form 2), 7P12 (DeNovoTIM13‐SB).
