# Supplementary material for: A newly introduced salt bridge cluster improves structural and biophysical properties of de novo TIM barrels
Source: Protein Sci. 2021 Dec 16;31(2):513–27. doi: 10.1002/pro.4249 (PMC8820119; doi:10.1002/pro.4249)
Supplement: Supplementary file 1 — Appendix S1: Supporting Information [file PRO-31-513-s001.docx]

# Supplementary material for:

**A newly introduced salt bridge cluster improves structural and biophysical properties of *de novo* TIM barrels**

Sina Kordes,^1†^ Sergio Romero-Romero,^1†^ Leonie Lutz,^1^ Birte Höcker^1*^

^1^ Department of Biochemistry, University of Bayreuth, 95447 Bayreuth, Germany.

† These authors contributed equally to the work.

* Corresponding author: Birte Höcker. Department of Biochemistry, University of Bayreuth, 95447 Bayreuth, Germany, e-mail address: [birte.hoecker@uni-bayreuth.de](mailto:birte.hoecker@uni-bayreuth.de)

**This file includes:**

- Supplementary Tables 1-4.
- Supplementary Figures 1-7.

# Supplementary Tables

**Supplementary Table 1. Amino acid sequences of parental DeNovoTIMs and salt bridge cluster variants.**


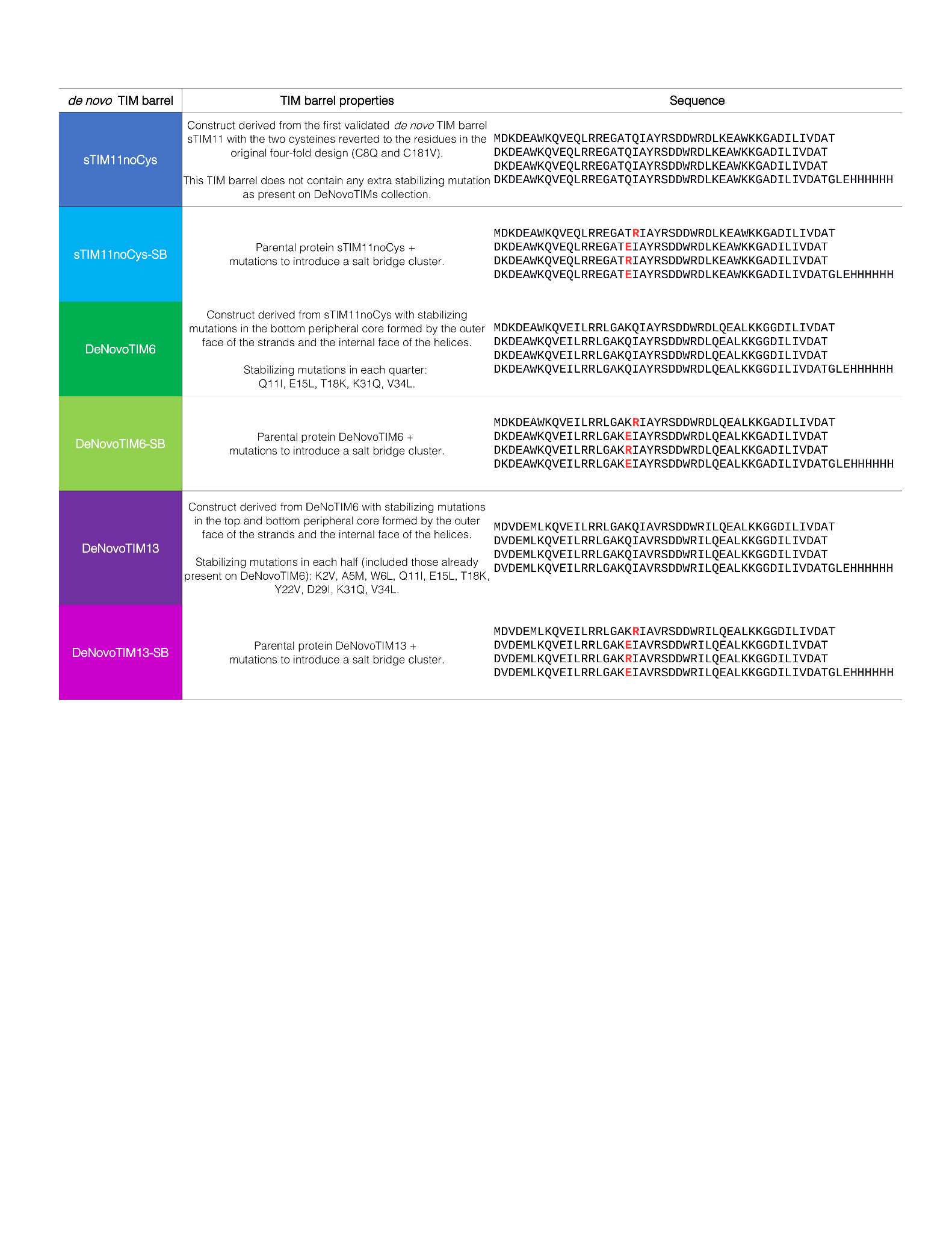


**Supplementary Table 2. Biochemical and biophysical properties of salt bridge cluster variants in comparison with the parental proteins.**

* Parameters reported in ref. 27.
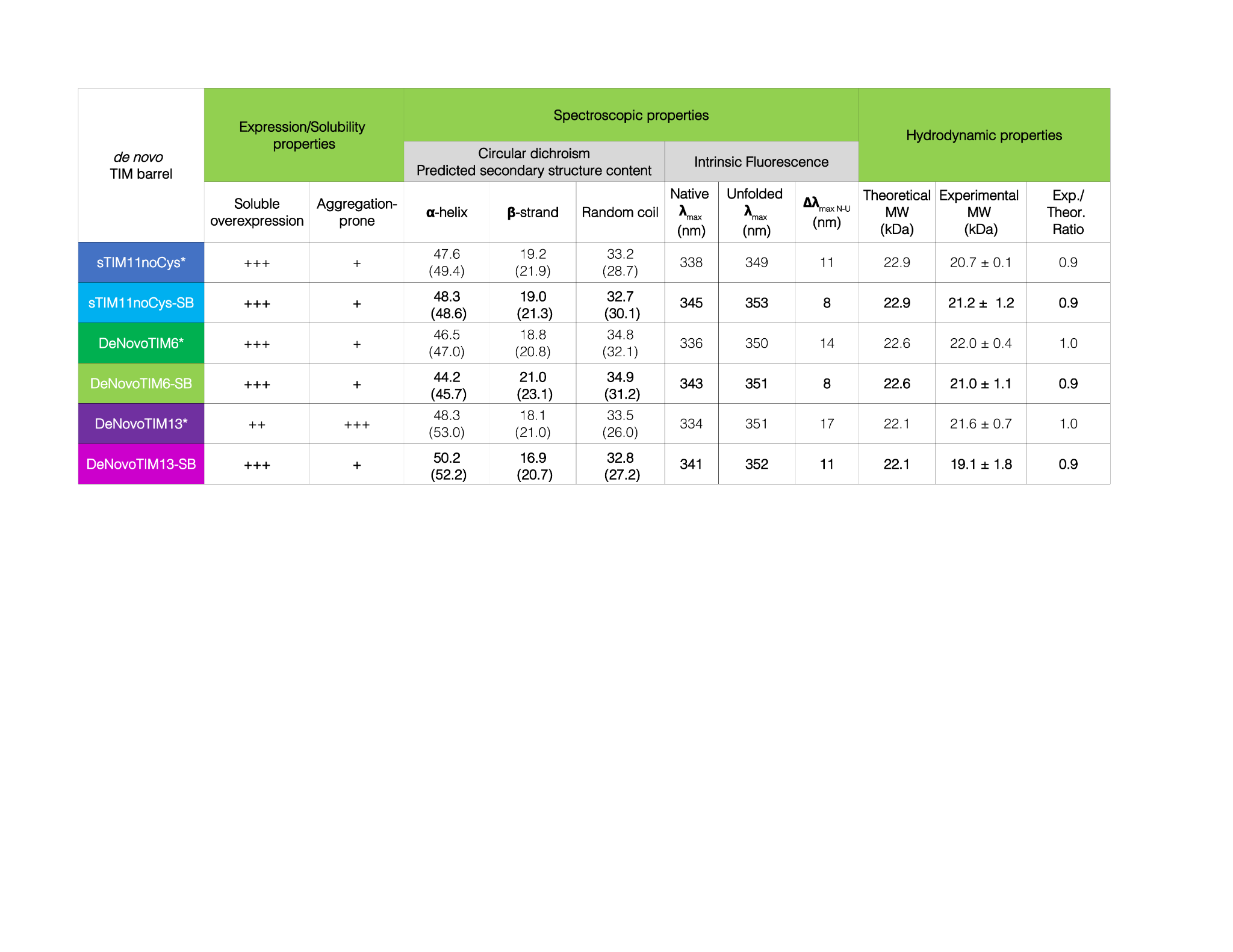


Numbers in brackets in the CD data are the calculated values from the three-dimensional structure.

# Supplementary Table 3. Crystallographic data collection and refinement statistics for the salt bridge cluster variants. Statistics for the highest resolution shell are shown in parentheses.


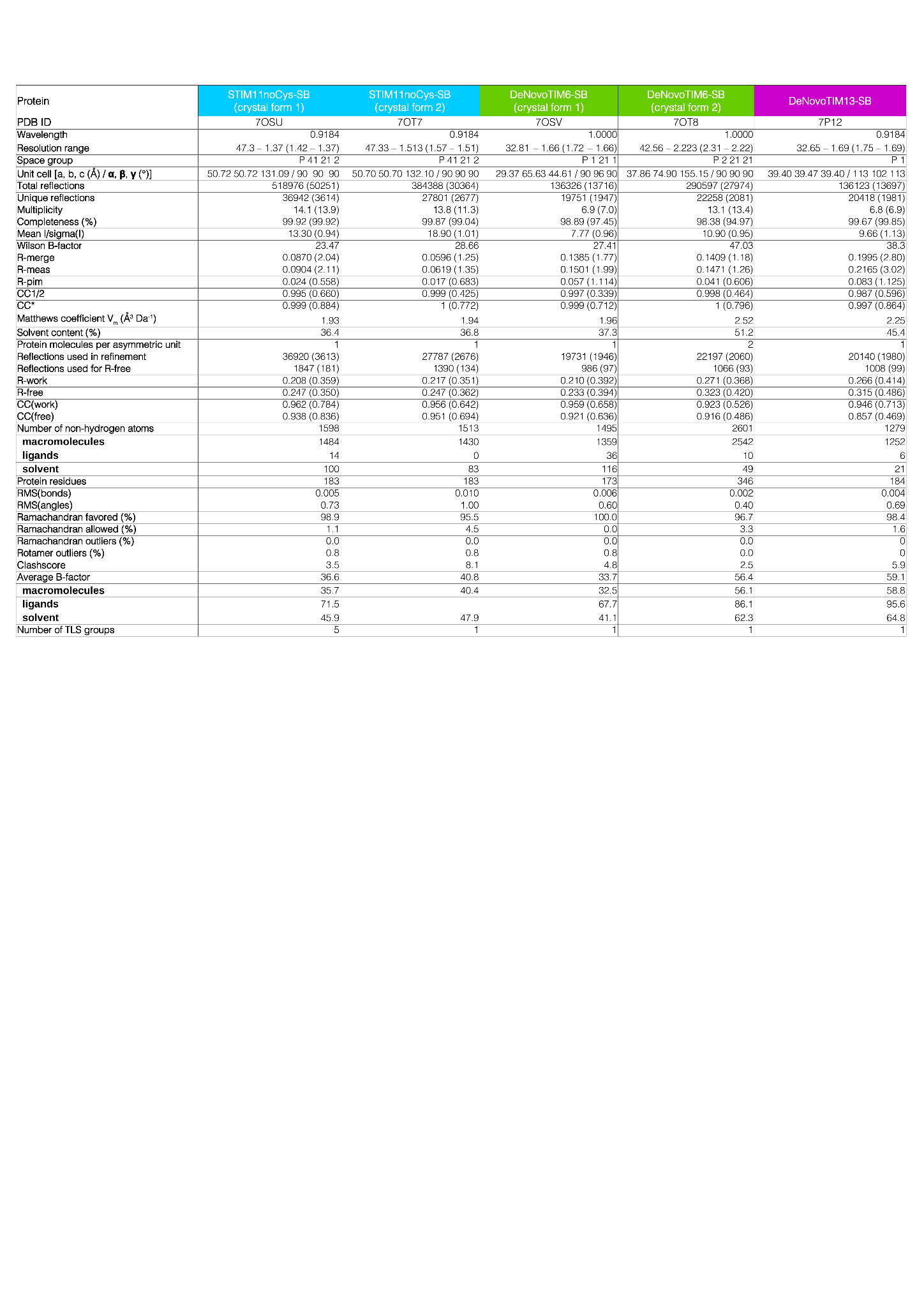


# Supplementary Table 4. Rosetta scores for the salt bridge cluster variants in comparison with the parental proteins.

#
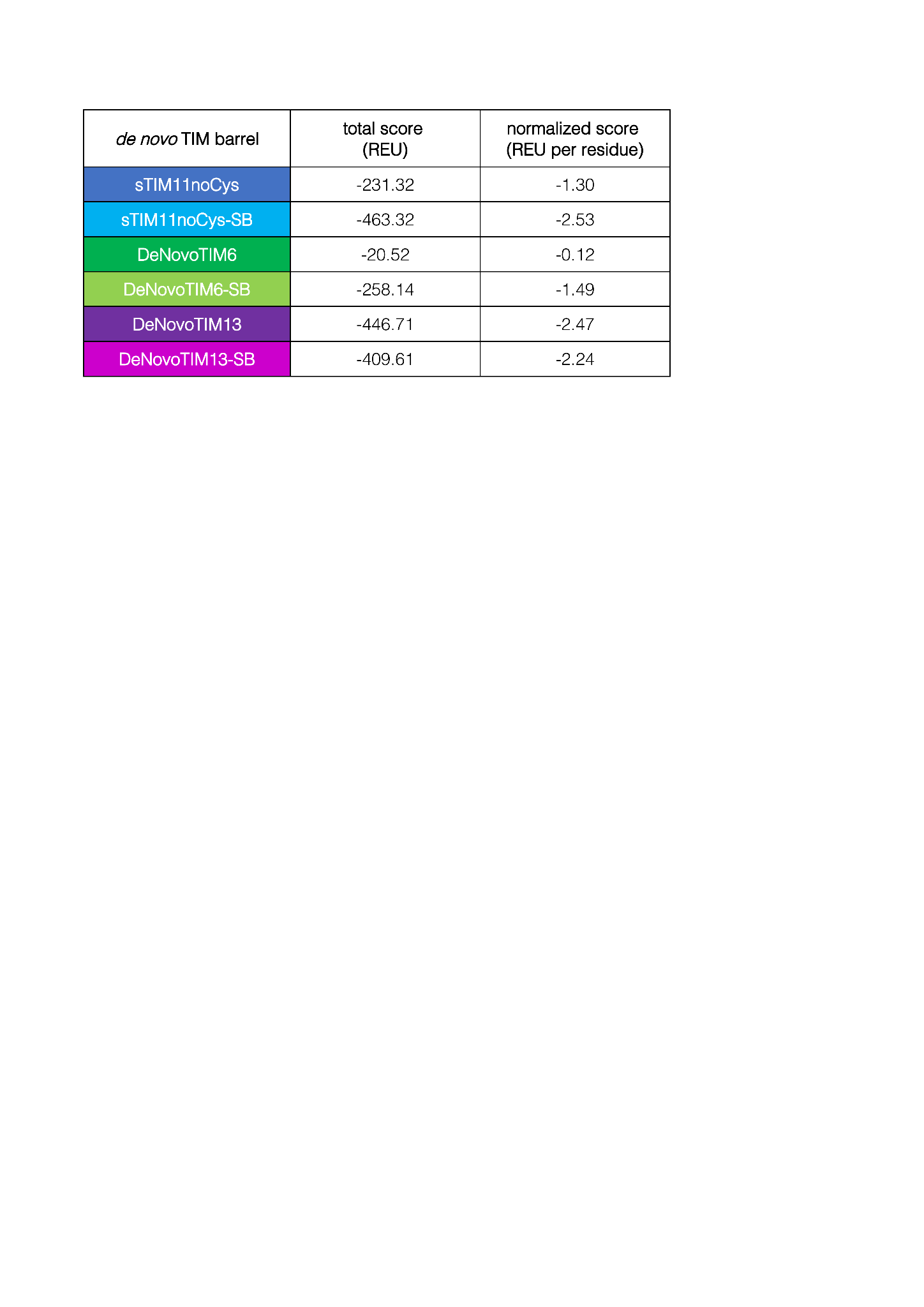


#

# Supplementary Figures


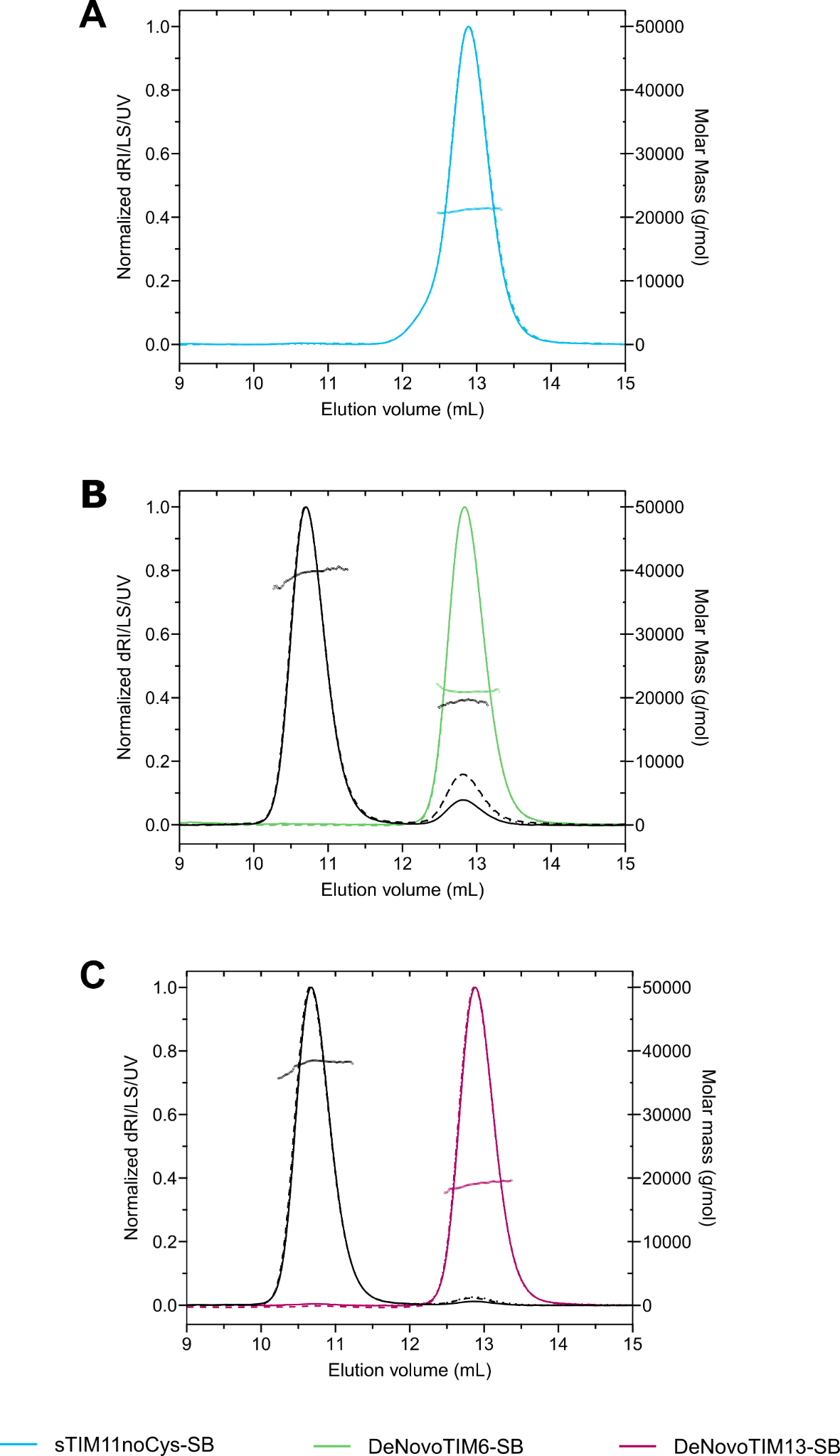


**Supplementary Figure 1. SEC-MALS of the salt bridge cluster variants**. **A)** Monomeric peak for sTIM11noCys-SB, **B)** Dimer (black lines) and monomer peak (green lines) for DeNovoTIM6-SB, **C)** Dimer (black lines) and monomer peak (pink lines) for DeNovoTIM13-SB. All plots show the elution volume versus either the normalized data for UV absorbance at 280 nm (UV, solid line), the differential refractive index (dRI, dashed line) and the multi-angle light scattering (LS, dotted line) (left axis), or the calculated molar mass (right axis, dot symbols). Values derived from the experiments are reported in the Supplementary Table 2. All experiments were performed in 35 mM sodium phosphate, 150 mM NaCl, 0.02% sodium azide, pH 8.


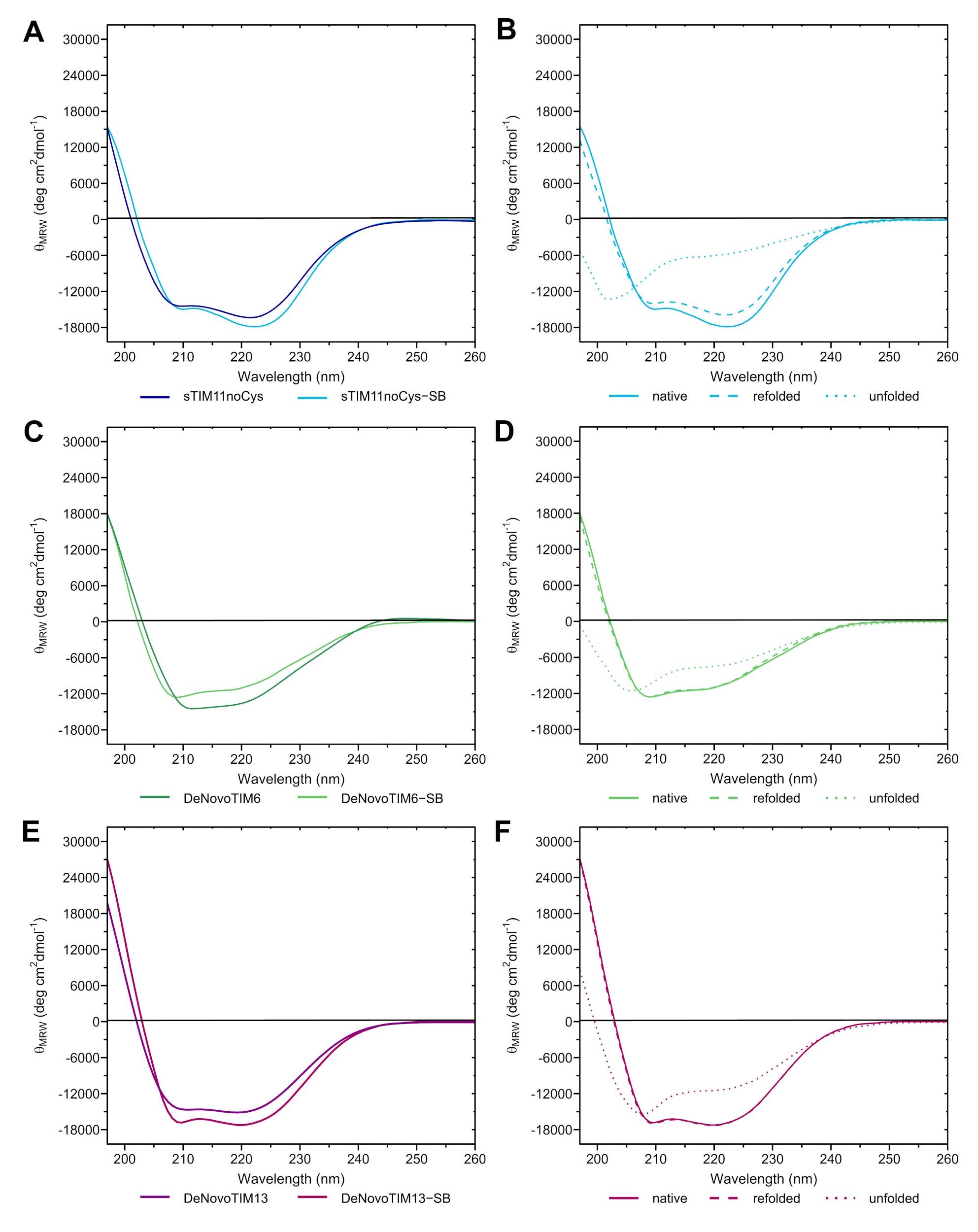


**Supplementary Figure 2. Far-UV CD spectra of the salt bridge variants**. The comparison between the parental proteins and the salt bridge variants in their native form are shown in panels **A)** (sTIM11noCys/sTIM11noCys-SB), **C)** (DeNovoTIM6/DeNovoTIM6-SB), and **E)** (DeNovoTIM13/DeNovoTIM13-SB). The experimental data for the native protein (solid lines), the unfolded protein at 95 °C (dotted lines), and the refolded protein after the T-melt (dashed lines) for the salt bridge variants are shown in panels **B)** (sTIM11noCys-SB), **D)** (DeNovoTIM6-SB), and **F)** (DeNovoTIM13-SB). All experiments were performed in 10 mM sodium phosphate pH 8.


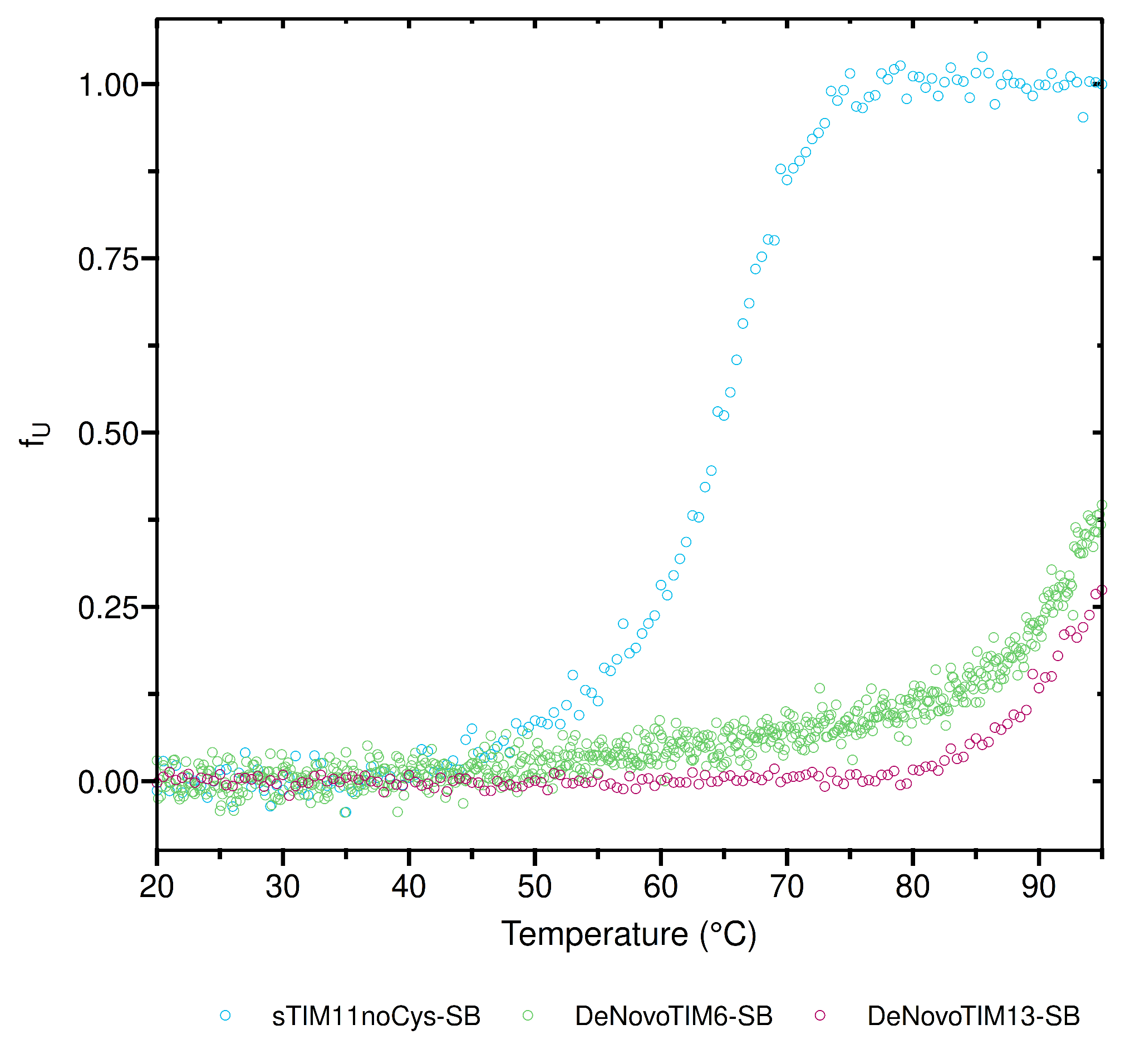


**Supplementary Figure 3.** **Far-UV CD T-melt of the salt bridge variants**. All experiments were collected following the CD signal at 222 nm with a protein concentration of 0.2 mg mL^-1^, a scan rate of 1.5 °C min^-1^, and 10 mM sodium phosphate pH 8.


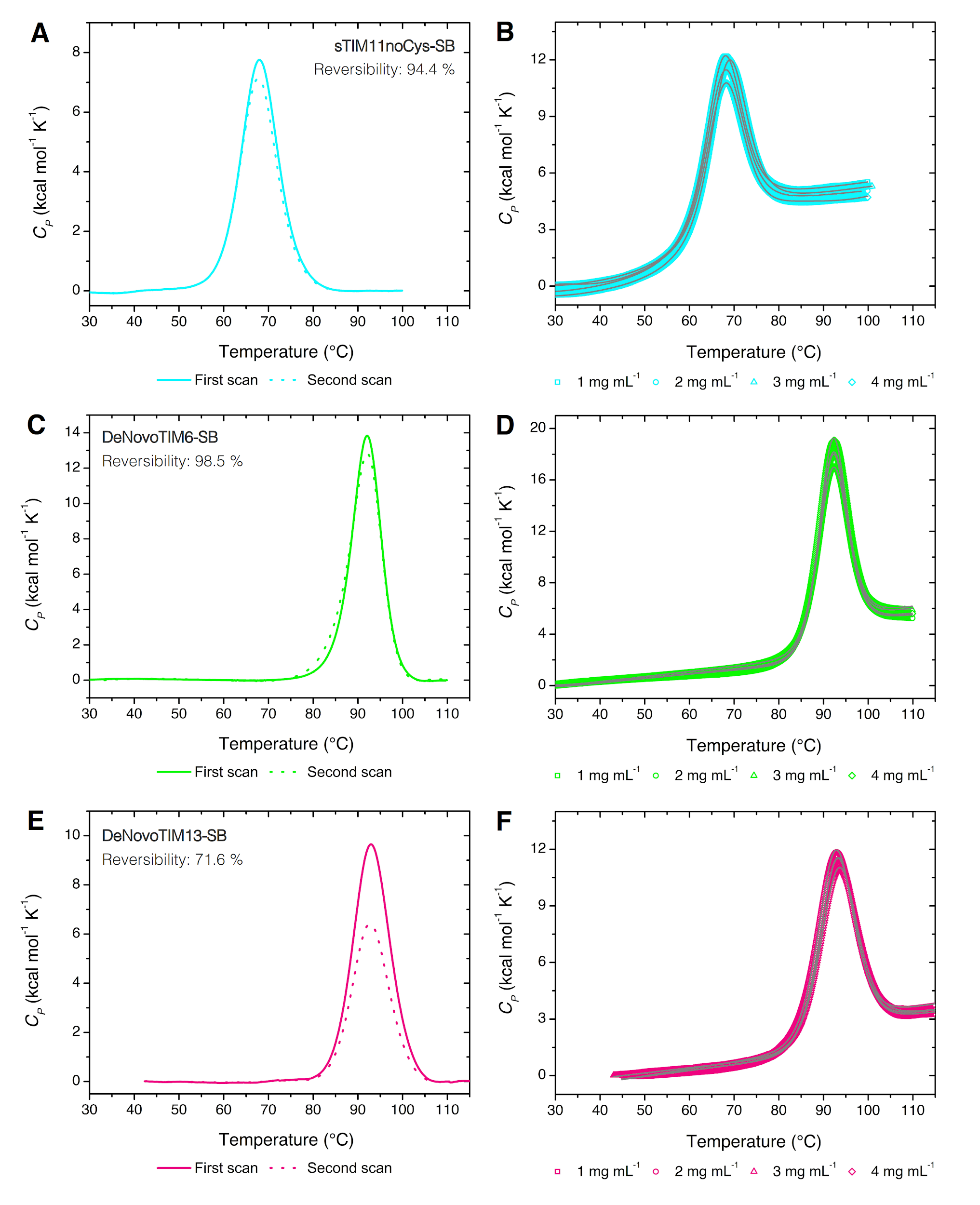


**Supplementary Figure 4.** **Thermal unfolding of the salt bridge variants followed by DSC**. Thermal unfolding reversibility was determined by the recovered area percentage by comparing the first and second scan (continuous and dotted lines, respectively in panels A, C, E). Thermodynamic parameters were calculated fitting the endotherms collected at different protein concentrations (open symbols) to a reversible two-state model (continuous lines in panels B, D, F). In panels A, C, and E, protein concentration was 1.0 mg mL^-1^. All scans were collected in 10 mM sodium phosphate pH 8.


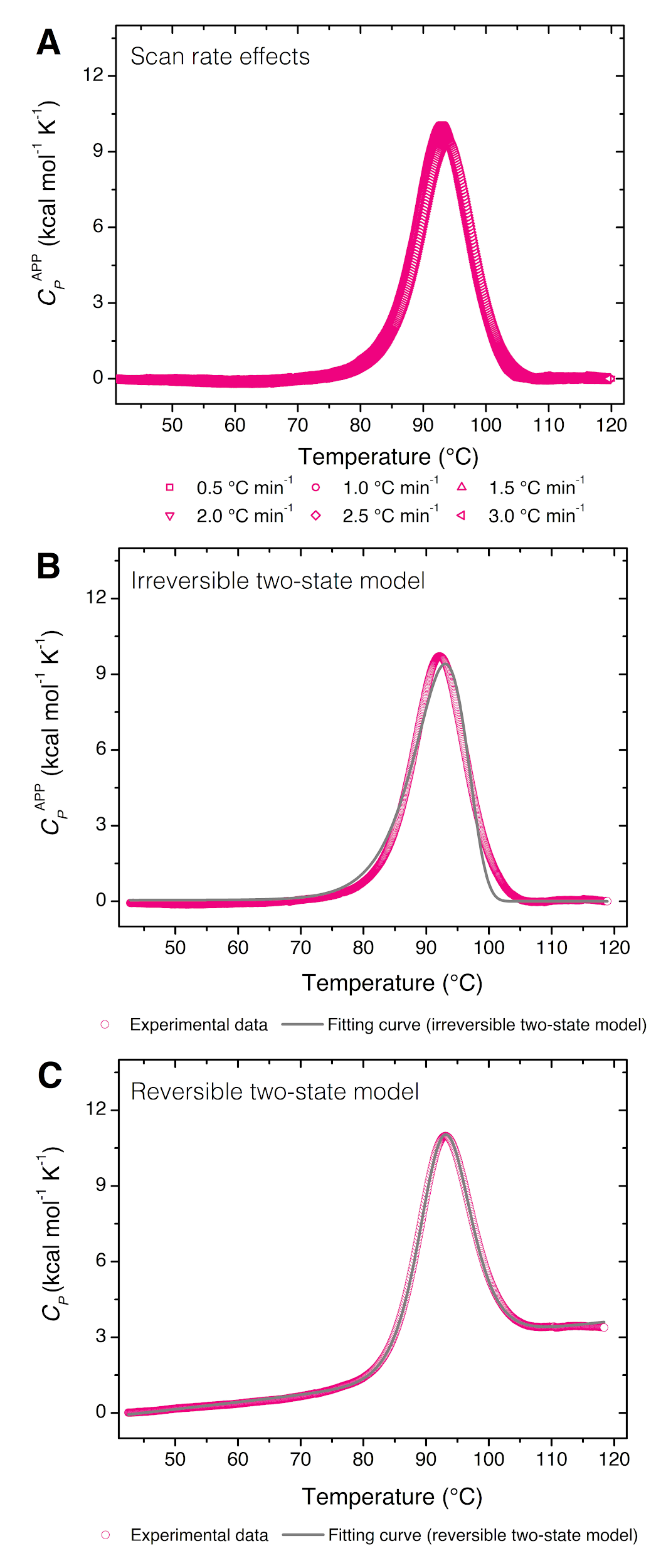


**Supplementary Figure 5.** **Thermal unfolding of DeNovoTIM13-SB followed by DSC. A)** Scan rate effects on the melting temperature. Endotherms were collected at scan rates from 0.5 to 3.0 °C min^-1^. **B)** Fitting example of an endotherm collected at 1.5 °C min^-1^ (symbols) fitted to the irreversible two-state model (continuous line). **C)** Fitting example of an endotherm collected at 1.5 °C min^-1^ (symbols) fitted to the reversible two-state model (continuous line). In all experiments protein concentration was 1 mg mL^-1^ in 10 mM sodium phosphate pH 8.


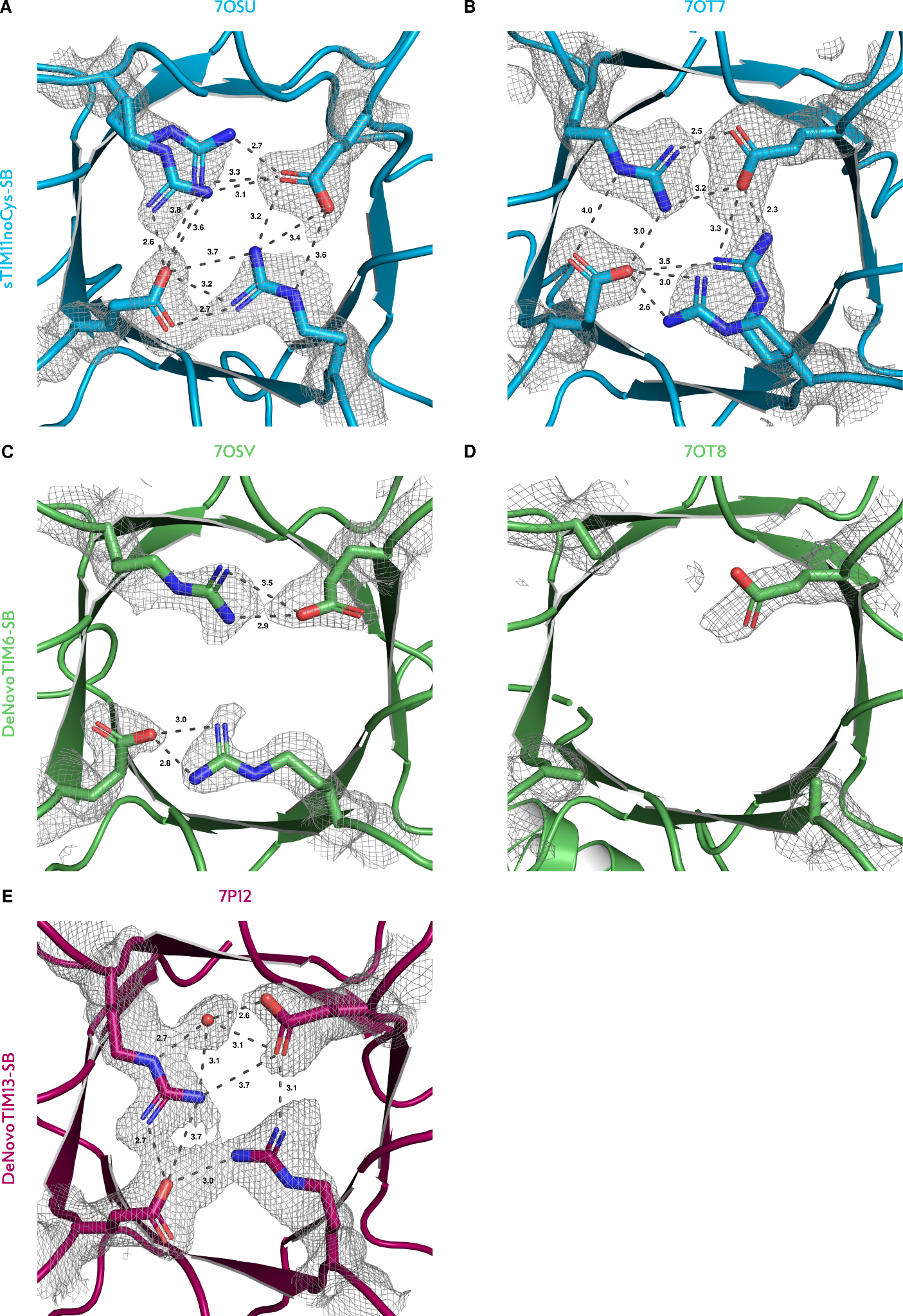


**Supplementary Figure 6.** **Comparison of the salt bridge interactions for all crystallography datasets.** Top panels show the comparison between the sTIM11noCys-SB datasets for the crystal form 1 **(A)** and crystal form 2 **(B)**. Middle panels indicate the comparison between the DeNovoTIM6-SB datasets for the crystal form 1 **(C)** and crystal form 2 **(D)**. Bottom panel shows the salt bridge cluster for the DeNovoTIM13-SB dataset **(E)**.


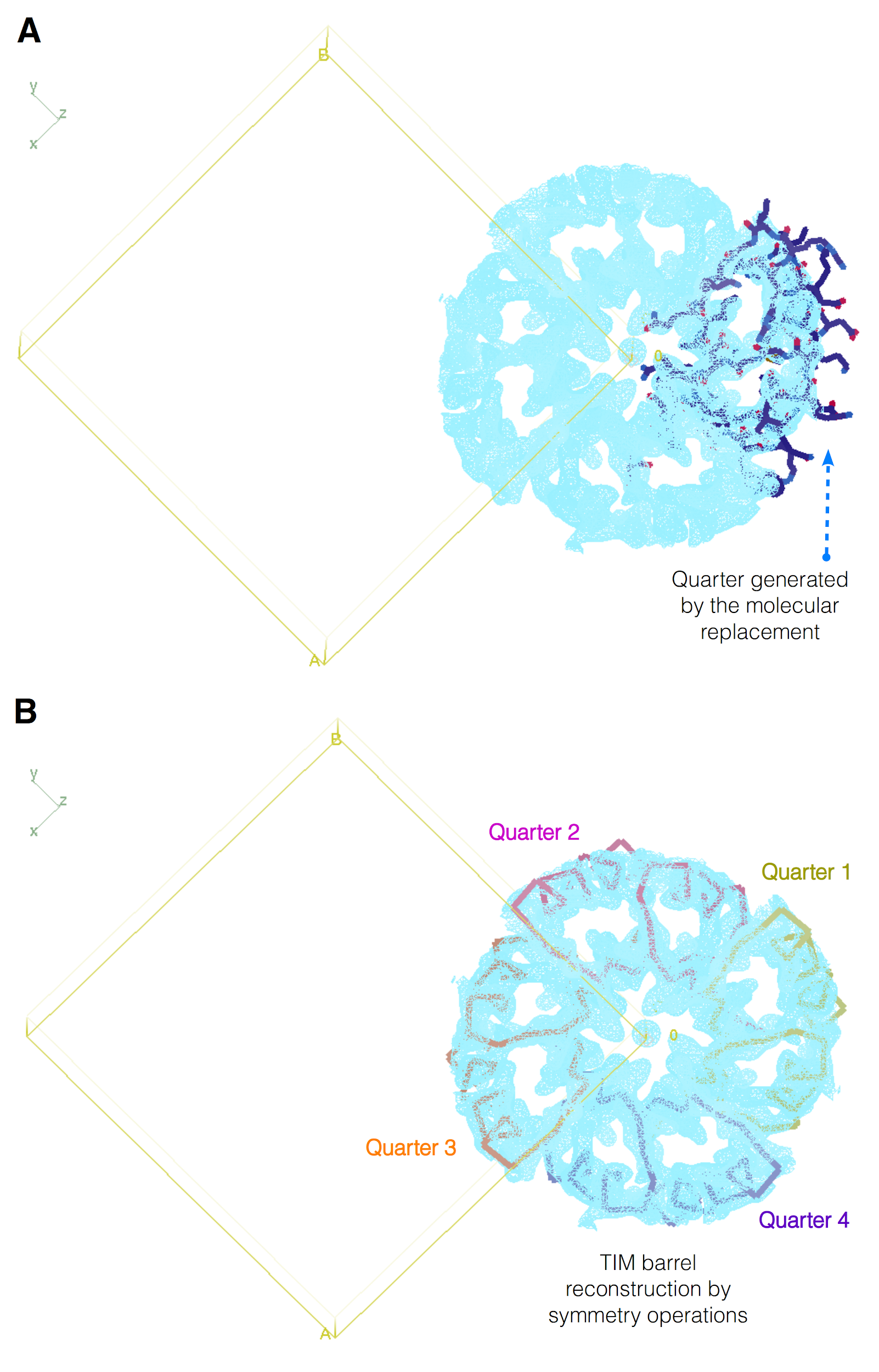


**Supplementary Figure 7.** **Molecular Replacement of DeNovoTIM13-SB structure in space group I4 (#79)**. **A)** TIM-barrel quarter generated by the molecular replacement processing of the data in this space group. **B)** TIM barrel reconstructed by using the corresponding symmetry operations. The four quarters are shown in different colors to highlight the proper formation of the barrel. For both panels, the unit cell is indicated in yellow.
